# Approved and Commercialized Antidiabetic Medicines (Excluding Insulin) in Seven European Countries—A Cross-Sectional Comparison

**DOI:** 10.3390/ph17060793

**Published:** 2024-06-17

**Authors:** Ana-Maria Atănăsoie, Robert Viorel Ancuceanu, Dušanka Krajnović, Magdalena Waszyk-Nowaczyk, Marcin Skotnicki, Dorota Tondowska, Guenka Petrova, Andrei Marian Niculae, Adriana-Elena Tăerel

**Affiliations:** 1 Department of Management and Pharmaceutical Marketing, Faculty of Pharmacy, “Carol Davila” University of Medicine and Pharmacy, 020956 Bucharest, Romania; 2 Department of Pharmaceutical Botany and Cell Biology, Faculty of Pharmacy, “Carol Davila” University of Medicine and Pharmacy, 020956 Bucharest, Romania; 3 Department of Social Pharmacy and Pharmaceutical Legislation, Faculty of Pharmacy, University of Belgrade, 11221 Belgrade, Serbia; 4 Pharmacy Practice and Pharmaceutical Care Division, Department of Pharmaceutical Technology, Poznan University of Medical Sciences, 60-806 Poznan, Poland; 5 Industrial Pharmacy Division, Department of Pharmaceutical Technology, Poznan University of Medical Sciences, 60-806 Poznan, Poland; 6 Community Pharmacy, 60-688 Poznan, Poland; 7 Department of Organization and Economy of Pharmacy, Faculty of Pharmacy, Medical University, 1000 Sofia, Bulgaria; 8 Department of Cellular, Molecular and Histology Biology, Faculty of Medicine, “Carol Davila” University of Medicine and Pharmacy, 050474 Bucharest, Romania

**Keywords:** diabetes medicines, authorized medicines, pharmacy price

## Abstract

Diabetes mellitus is a complex, multifactorial, progressive condition with a variety of approved therapeutic options. The purpose of this study was to offer an overview of the authorized antidiabetic medicines (excluding insulin) compared with marketed products in seven European countries. Data were obtained from primary sources, including the websites of national authorities and directly from specialists in the countries of interest. The range of marketed medicines compared with the authorized group was assessed in terms of active pharmaceutical ingredients (>60% in Bulgaria, France, Serbia), brand names (>70% in Bulgaria, the Czech Republic, Romania, Serbia, Spain), pharmaceutical forms (>60% in all countries), strengths (>60% in Bulgaria, the Czech Republic, Romania, Serbia, Spain), marketing authorization holder (≥50% in all countries) and the status of medicine. Spain was found to have the highest number of products based on most of these attributes. Over 90% of authorized medicines had a pharmacy price in Serbia. Regarding the newer class of GLP-1 receptor agonists, a retail price for all approved substances was available in Bulgaria, Romania, Serbia, and Spain. Only one brand name with one concentration was found available for some agents, being susceptible to drug shortages: glibenclamide (Romania, Serbia, Spain), glipizide (the Czech Republic, Poland, Romania, Spain), glisentide (Spain), acarbose (the Czech Republic), sitagliptin (Bulgaria, Poland), vildagliptin (the Czech Republic, Poland) and saxagliptin (the Czech Republic, France, Romania, Serbia). An overview of the national and international therapeutic options may allow competent authorities and health professionals to take rapid measures in case of supply problems or health crises.

## 1. Introduction

Diabetes mellitus (DM) is a chronic, progressive disease associated with serious disability, reduced quality of life, and high rates of mortality. Diabetes affects one in ten adults globally, imposing a high burden on national healthcare systems, patients, and their families [1,2,3,4]. With a predicted growth of 46% of cases globally in 2045 and a smaller percentage in the European region (13%), it is regarded as one of the most threatening non-communicable illnesses of the 21st century. The International Diabetes Federation (IDF) reported that the age-adjusted (20–79 years) estimated prevalence of diabetes in Europe was 7.0% (2021), with higher values in 14 countries (for example, Bulgaria (7.4%), the Czech Republic (7.1%), Serbia (9.1%), Spain (10.3%)) compared with countries such as France (5.3%), Romania (6.5%), and Poland (6.8%). It was estimated that by 2045, it will see an increase in the European Region (8.7%), along with all countries: Bulgaria (8.7%), the Czech Republic (8.6%), Serbia (10.9%), Spain (12.7%), France (6.5%), Romania (7.5%), and Poland (8.5%) [5].

Depending on the type of diabetes (type 1, type 2), uncontrolled diabetes may lead to serious acute (ketoacidosis, hypoglycemia, lactic acidosis) and chronic complications (cardiovascular disease, nephropathy, retinopathy, foot ulcer, kidney failure) [6]. The UK Prospective Diabetes Study showed that maintaining glycemic control would lead to better treatment outcomes and fewer complications [7].

Over 90% of individuals with diabetes have type 2 diabetes mellitus (T2DM), making it the most prevalent form of the disease [5]. The pharmacological treatment for T2DM has a significant financial impact on national healthcare systems and patients [8], as the cost of several antidiabetic oral medicines is not fully covered by national insurance funds. T2DM management has become very complex. In recent years, research trends have been oriented towards a patient-centered approach and individualized therapeutic plans, in terms of therapy management and drug formulation [9,10,11]. According to international diabetes associations, choosing the appropriate pharmaceutical intervention is not considered sufficient to obtain successful health outcomes [12]. Therefore, medical professionals should address not only clinical conditions but also take into account patient characteristics, needs, satisfaction, and lifestyle conditions [13]. However, in order to select an individualized treatment, there should be a wide range of pharmaceutic options in terms of active substances, concentrations, routes of administration, commercial names, or prices. 

Before reaching patients, medicines must be authorized through one of the established European Union (EU) procedures (national, mutual recognition, decentralized, and centralized) [14]. The centralized procedure (involving evaluation by the European Medicines Agency) is mandatory for human medicines based on a new active substance when the condition for which it is intended is diabetes [15]. After a marketing authorization is granted, companies usually need to submit a pricing and, optionally, a reimbursement application to national authorities [14] in order to make the medicine available for patients to purchase from pharmacies. Sometimes, both generic and innovative medicine manufacturers cannot accept the price proposed or imposed by the relevant authorities and choose not to market the medicine (or certain presentation forms of that medicine) in that country. In this light, in order to gain more knowledge about the availability of antidiabetic agents at the national level, it is important to have a realistic perspective on authorized and marketed pharmaceuticals in different European countries.

In recent years, drug shortages have become a significant problem worldwide, which has been a challenge for healthcare professionals and has substantially affected patient care [16]. International and national regulations were adopted or updated to prevent, minimize, and manage the impact of disruptions in medicine. One domain of interest for the European Medicines Agency (EMA) has been the availability and accessibility of medicines, especially after the COVID-19 pandemic. In December 2016, EMA and the Heads of Medicines Agencies (HMA) established a joint task force on the availability of authorized medicines for human and veterinary use (TF AAM). There are three situations that can lead to a reduced quantity of medicines as described by the task force: lack of authorized medicines, the absence of authorized medicines on the market, and supply chain issues [17]. In addition to this, in November 2020, the European Commission proposed a new pharmaceutical strategy, with the improvement of accessibility and affordability of medicines, as well as securing supply chains, as two key areas of interest [18]. It was mentioned that a new authority named HERA (Health Emergency Preparedness and Response Authority), established in September 2021 as a general directorate within the European Commission, was created to ensure a prompt and appropriate response to pandemics and similar health threats of large dimensions, including inter alia procurement of necessary medicinal products [19]. Around the same time, the EU authorities adopted Regulation (EU) 2022/123 of the European Parliament and the Council on 25 January 2022 on a reinforced role for the European Medicines Agency in crisis preparedness and management for medicinal products and medical devices [20]. In addition, the European Commission put out a proposal on 26 April 2023 to revise the current pharmaceutical legislation, mainly Regulation 726/2004 and Directive 2001/83/EC. Within this initiative, key priority domains are access to affordable medicines, availability of medicines, and continuous pharmaceutical supply for patients [21].

Multiple studies have been published where the authors analyzed patterns of diabetes treatment [22,23,24], medicines consumption [25], or clinical practice adherence to national and international guidelines [26]. However, the relationship between the total number of authorized medications and pharmaceuticals available to patients on the national markets (leaving aside supply problems considerations), as well as the proportion of medicines that are available out of the total number of authorized products, have been less explored.

The objective of this study was to perform a comparative analysis of the ranges of approved and available antidiabetic agents (excluding insulins) in seven European countries. The availability of pharmacy retail prices was used as an imperfect proxy for the availability of the products on the market. A second objective was to assess the features of the marketed treatments for type 2 diabetes in those countries and to identify medicines susceptible to being associated with more serious consequences in case of a drug shortage.

## 2. Results

### 2.1. General Attributes of the Range of Antidiabetic Medicines

By comparing the range of authorized medicines to the subgroup of medicines for which a retail price was available (Table 1) for each country (individually), we found that Serbia had the highest percentage of drugs with a pharmacy price (91.0%, 193 products with price from 212 authorized medicines). Spain trailed behind it in the second place with only 46.3%. Serbia possesses the highest proportion of marketed brand names, with a prevalence of 90.7%. Bulgaria follows behind with 71.8%, while France, Romania, and Spain exhibit corresponding percentages of 60.4%, 58.3%, and 57.7%. In the majority of the studied countries, more than 70% of the authorized active substances have at least one product with a retail price. In three countries (Bulgaria, Romania, Serbia), all authorized pharmaceutical forms have a pharmacy price, with over 60% in the remainder of the countries.

### 2.2. Observations

**Figure 1:**
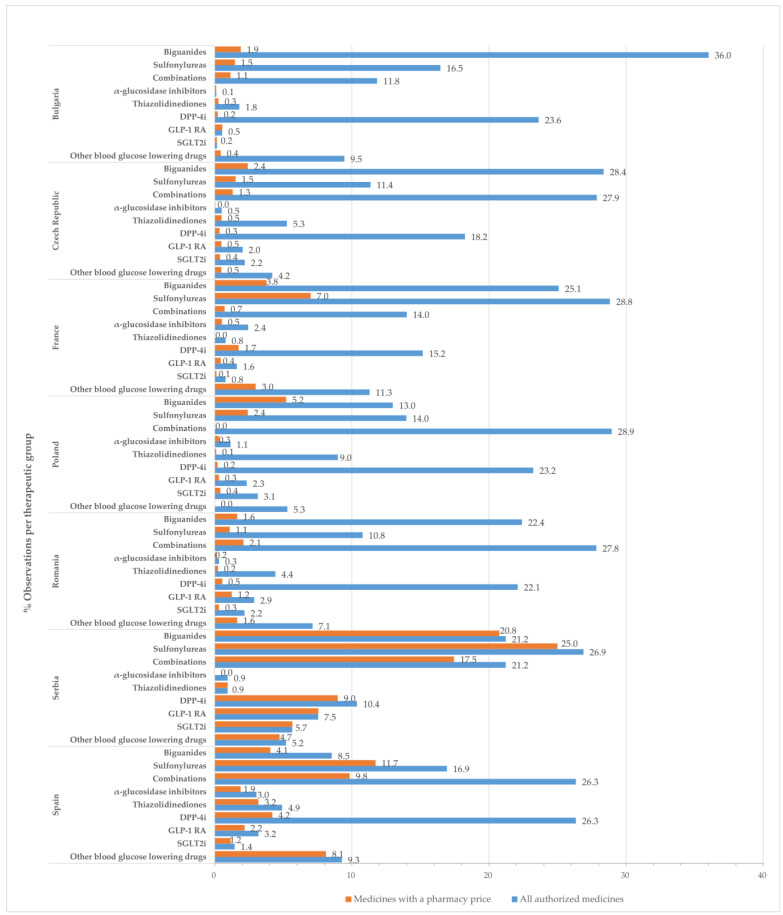
Percentage of observations (%) for all approved medicines and those with a pharmacy price, in relation to the total number of medicines with marketing authorization by therapeutic class and country.

In this study, an “observation” corresponds to a presentation form of a medicinal product. Figure 1 displays the percentage of observations for medicines with approved prices relative to the total number of authorized medicinal products at the therapeutic group level by country.

Drugs belonging to the groups of biguanides (metformin), sulfonylurea derivatives, dipeptidyl peptidase 4 (DPP-4) inhibitors, and combinations groups occupied most positions in the list of all authorized medicines and medicines with approved prices.

### 2.3. Active Substances (INNs)

Nine therapeutic groups (detailed in the Section 4) were identified as authorized, including 27 active substances (biguanides-1; sulfonylurea-6; alpha-glucosidase inhibitors-1; thiazolidinediones-1; dipeptidyl peptidase 4 (DPP-4) inhibitors-5; glucagon-like peptide-1 (GLP-1) analogues-5; sodium-glucose co-transporter 2 (SGLT2) inhibitors-5; other blood glucose-lowering drugs-3) and 17 oral fixed combinations of substances, based on data from all participating countries. Among these, no pharmaceutical products containing sotagliflozin or the combination metformin + saxagliptin + dapagliflozin were available at retail prices in the included countries. The individual situation for each country is available in Tables S1 and S2.

**Figure 2:**
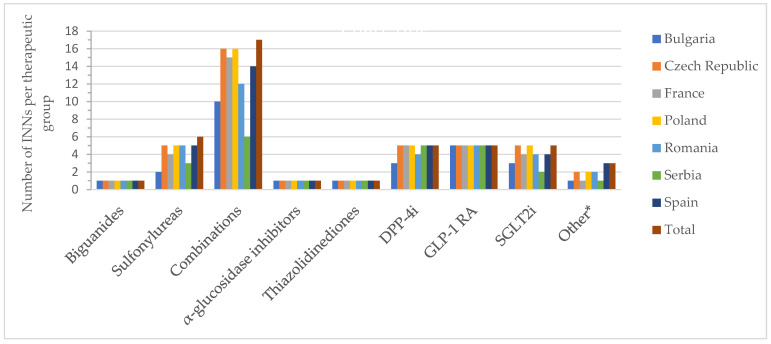
Number of authorized INNs (out of all approved INNs of a therapeutic group from all countries) by therapeutic group and country; * other = other blood glucose-lowering drugs.

**Figure 3:**
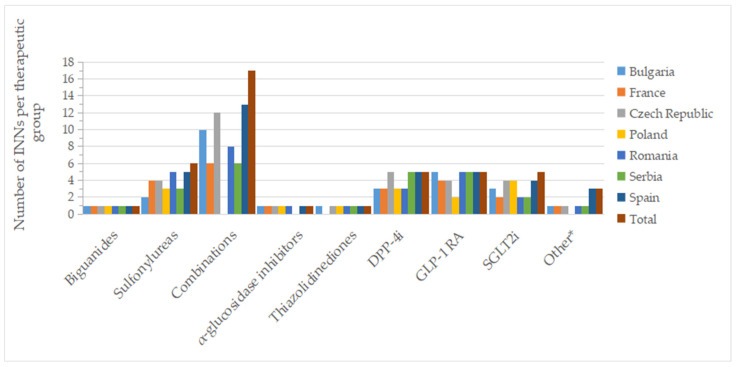
Number of INNs with a pharmacy price (out of all approved INNs of a therapeutic group from all countries) by therapeutic group and country; * other = other blood glucose-lowering drugs.

Figure 2 and Figure 3 show the number of INNs for all approved drugs and those with a price out of the total number of authorized substances and combinations from all countries analyzed. Among the therapeutic groups, medicines containing metformin (biguanides), acarbose (α-glucosidase inhibitors), and pioglitazone (thiazolidinediones) were authorized in all countries. However, acarbose seems not to be marketed in Serbia (it did not appear on the list of drugs with wholesale prices in Serbia), as was the case for pioglitazone in France. For sulfonylurea derivatives, among the 6 INNs (glibenclamide, glipizide, gliquidone, gliclazide, glimepiride, glisentide), 5 INNs (83.3%) were authorized in the Czech Republic, Poland, Romania, and Spain, with gliquidone not authorized in Spain, and glisentide not authorized in the other three countries, respectively. In the Czech Republic, glibenclamide, although authorized, is not marketed (no retail price is available) (Table S1). Authorized medicines based on the five active substances from the SGLT2i group (dapagliflozin, canagliflozin, empagliflozin, ertugliflozin, sotagliflozin) were well represented in Poland and the Czech Republic (100%, 5 INNs), followed by France, Romania and Spain (80%, 4 INNs). In France, Romania, and Serbia, only products based on dapagliflozin and empagliflozin were marketed (40%); additionally, canagliflozin was available in Bulgaria (60%), whereas in the Czech Republic and Spain, four substances were marketed (80%, excluding sotagliflozin).

### 2.4. Brand Names

**Figure 4:**
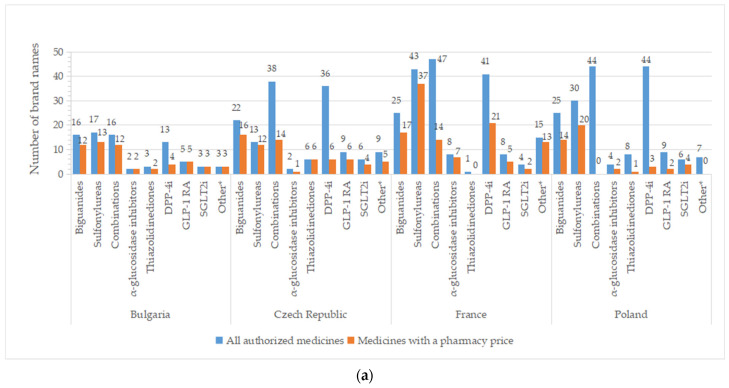

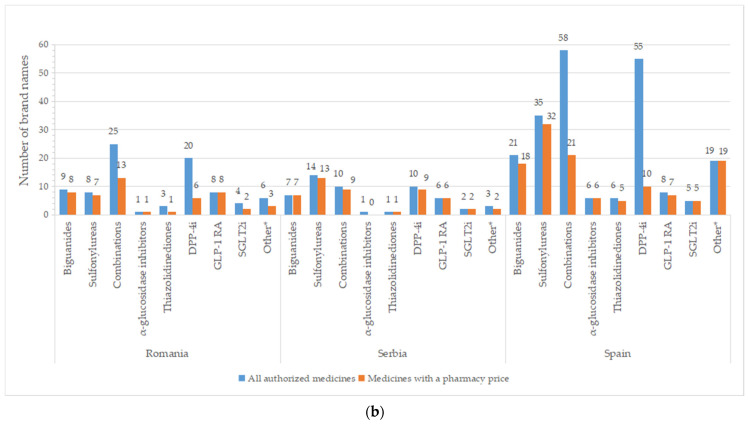
Total number of brand names for all diabetes medicines with marketing authorization and with a pharmacy price per therapeutic group and country: (**a**) Bulgaria, the Czech Republic, France, Poland; (**b**) Romania, Serbia, Spain. * Other = other blood glucose-lowering drugs.

A high number of brand names for all authorized drugs was reported in most of the countries, especially for groups containing older molecules (metformin, sulfonylurea derivates), but also for DPP-4i and oral fixed-combinations groups (Figure 4). A list with the number of brand names by INN and country is available in Table S2. Important differences between all pharmaceutical products and those with price were registered for DPP-4i (except for Serbia), fixed combinations (only 36.8%—the Czech Republic, 29.8%—France, and 36.2%—Spain from authorized commercial names had price). An overview of common brand names is presented in Tables S3 and S4.

There are two or more brand names marketed (having a price) for each INN for the majority of therapeutic groups (Table 2 and Table S2). Only one brand name is available for acarbose in the Czech Republic and Romania; the same holds true for pioglitazone in Poland, Romania, and Serbia. Several active substances from the DPP4-I class have only one brand name with a price: sitagliptin (Bulgaria, Romania, Poland), vildagliptin (the Czech Republic, Poland), and linagliptin (Bulgaria, the Czech Republic, Poland, Serbia, Spain; no drug in the rest of the countries). Repaglinide was the only representative from the A10BX group (other blood glucose-lowering drugs, excluding insulins) in the majority of the studied countries, found in a different number of brand names (Bulgaria and Romania—3, the Czech Republic—5, France—13, Serbia—2, Spain—17). In Spain, two other active substances were observed for the A10BX class, guar gum and nateglinide, with one brand name for each substance.

### 2.5. Type of Medicine

Medicines based on active substances from GLP-1 RA and SGLT2i classes (alone or in combination with other agents) are under data exclusivity or patent protections; therefore, generic products are not authorized. For the majority of therapeutic groups, the innovator pharmaceutical products (the reference medicinal product in the EU law sense) for every active substance were (still) authorized in most countries, with a small number of exceptions for INNs from sulfonylureas (glibenclamide—Romania), oral fixed-combinations groups (pioglitazone + metformin − Bulgaria, metformin + vildagliptin − Serbia), acarbose (the Czech Republic), pioglitazone (Bulgaria, Serbia) and other blood glucose-lowering drugs (repaglinide—Bulgaria, Romania, Serbia). Metformin has one innovative medicine in Spain and France and two in the rest of the studied countries (similar names, but with two pharmaceutical forms) (Table 3 and Table S5). No authorized generic medicines were available for α-glucosidase inhibitors (Romania, Serbia), thiazolidinediones (France), some sulfonylureas (the Czech Republic, Poland, Romania, Serbia, Spain), some oral fixed combinations (in all countries) and some DPP-4i (saxagliptin—generic approved only in France, alogliptin—without generic in all countries, linagliptin—Bulgaria, Romania, Serbia). Generic medicines based on metformin had many authorized representatives: 5 (Serbia), 7 (Romania), 14 (Bulgaria), 20 (the Czech Republic, Spain), 23 (Poland), and 24 (France). Prices for innovator medicines were not available for some INNs from the sulfonylureas group (glibenclamide—Romania, glipizide—Poland), DPP-4i (vildagliptin—Poland, saxagliptin—Serbia), and pioglitazone (Poland, Romania) (Table 4 and Table S6). Repaglinide was the only representative from the other blood glucose-lowering drugs group in the majority of the studied countries, while in Spain two additional active substances were available: guar gum and nateglinide. Among the marketed products, no generics were found within the sulfonylureas group (except Bulgaria), and, among oral fixed combinations, the DPP-4i group and for acarbose (Romania).

### 2.6. Pharmaceutical Forms

**Figure 5:**
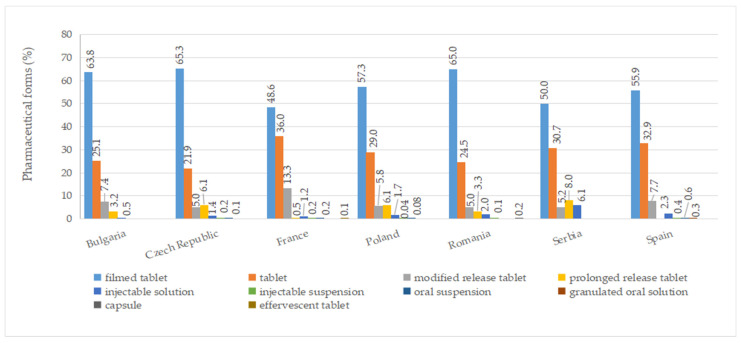
Pharmaceutical forms (%) of all authorized diabetes medicines by country.

**Figure 6:**
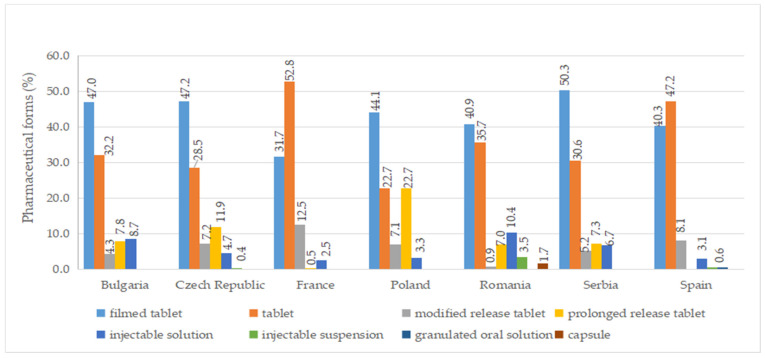
Pharmaceutical forms (%) of diabetes medicines with a pharmacy price (out of all medicines having a price) by country.

A total of ten pharmaceutical forms were identified for authorized medicines (Table S7). Two dosage forms (oral suspension and effervescent tablet) were apparently not marketed (no price available). Among the countries included in this study, France had the most diverse range of pharmaceutical forms for approved medicines, with a total of eight. This was followed by the Czech Republic, Poland, Romania, and Spain, each of which had seven different authorized pharmaceutical forms. As seen in Figure 5, the most commonly authorized pharmaceutical form was film-coated tablets, accounting for over 40% of the total, followed by conventional tablets, representing more than 20%. Capsules were only authorized in Romania, while effervescent tablets were exclusively approved in France. Granulated oral solution formulations were found solely in Spain. When considering medicines with a price, conventional tablets (>20%), film-coated tablets (>30%), injectable solutions, and modified-release tablets were available in all countries (Figure 6).

**Figure 7:**
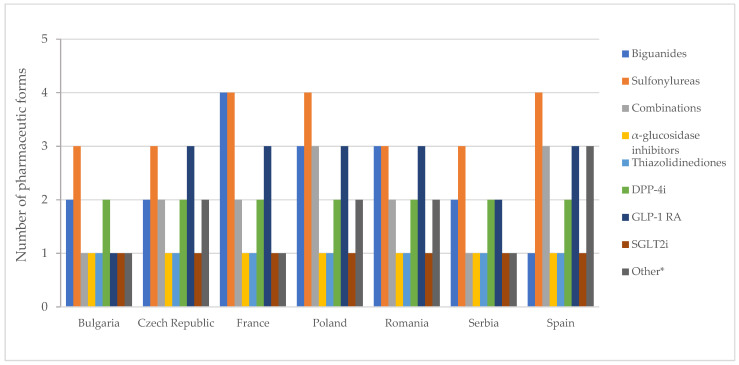
Number of pharmaceutical forms of all diabetes medicines with a marketing authorization by therapeutic group. * Other = other blood glucose-lowering drugs.

The number of authorized pharmaceutical forms by class is shown in Figure 7. In most countries, authorized medicines containing metformin were available in the form of film-coated tablets and prolonged-release tablets (Table S7). Exceptions to this finding were noted in Spain, where only film-coated tablets are authorized, and in Poland and Romania, where conventional tablets are also approved. In France, there is a medicinal product based on metformin, formulated as effervescent tablets.

In France, Poland, and Spain, medicines based on sulfonylureas were formulated as immediate-release, modified, and prolonged-release solid forms and as an oral suspension. Acarbose and pioglitazone were exclusively authorized as film-coated tablets, while SGLT2 inhibitors were universally authorized as tablets across all countries.

**Figure 8:**
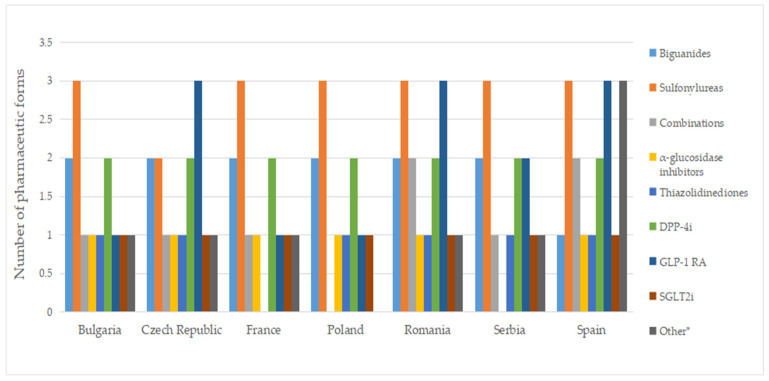
Number of pharmaceutical forms of diabetes medicines with a pharmacy price, by therapeutic group. * Other = other blood glucose-lowering drugs.

Figure 8 displays no differences between authorized and marketed pharmaceutical forms in Bulgaria and Serbia at the therapeutic group level (except for acarbose, which is not marketed in Serbia).

### 2.7. Strengths

**Figure 9:**
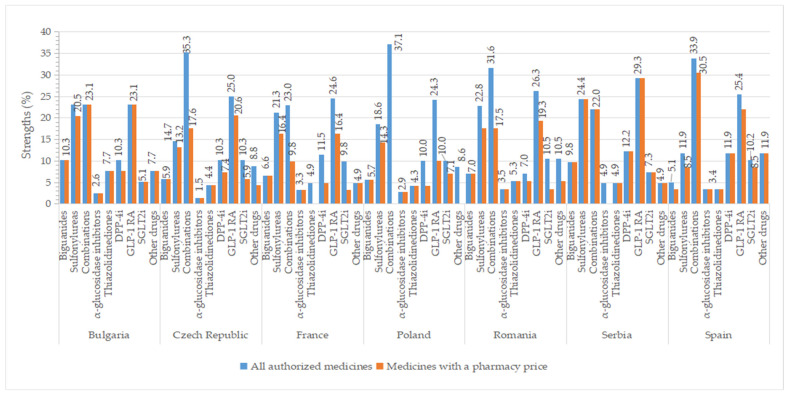
Percentages of strengths (out of the total number of authorized strengths in each country) for all authorized antidiabetic medicines and those with pharmacy prices by therapeutic group and country.

Higher percentages of authorized strengths were observed for GLP-1 agonists (>23%), oral fixed combinations (≥22%), and sulfonylureas (>11%) in all studied countries, whereas smaller percentages of strengths for groups represented by only one active substance like α-glucosidase inhibitors (<4%) and thiazolidinedione (<8%) (Figure 9). The number of approved strengths per INN is listed in Table S8. All four strengths authorized for metformin were commercialized in all countries, except Spain (where from three strengths, only two had a price). In Serbia, all authorized concentrations had a price (except for acarbose, which is unavailable, and saxagliptin, for which one of the two concentrations had a price). We found a difference of at least two strengths between all authorized medicines and those with a price for some representatives from sulfonylureas (the Czech Republic, France, Romania), combinations (except Bulgaria, Serbia), DPP-4 inhibitors (except Romania, Serbia, Spain), GLP-1 agonists (except Bulgaria, Romania, Serbia), SGLT2 inhibitors (France, Romania), and other blood glucose-lowering drugs (the Czech Republic, Romania).

Table 5 shows the distribution of strengths for medicines with a price per active substance across the studied countries. 

### 2.8. Marketing Authorization Holder

The first 10 MAHs selected by the number of INNs and commercial names were presented in Tables S9 and S10. AstraZeneca and Krka had antidiabetic medicines authorized in all studied countries (except KrKa in Bulgaria).

## 3. Discussion

In this study, we intended to provide a better understanding of the range of authorized and available antidiabetic drugs (having a pharmacy price) in seven selected European countries. Not all authorized active substances were available on the market for patients in all countries surveyed, except Bulgaria. Spain had the highest number of medicines with a pharmacy price in terms of brand names (123), INNs (25 active substances and 13 oral fixed combinations), MAHs (44), and strengths (51). A study performed from 2006 to 2016 in eleven countries showed that only in Spain were all active substances from the new therapeutic classes—GLP-1 receptor agonists and SGLT-2 inhibitors—commercialized [27]. In this study, five substances from GLP-1 agonists were approved in all countries, one of them (lixisenatide) not being available on the market in the Czech Republic, France, and Poland. Only medicines based on dulaglutide and semaglutide were commercialized in Poland. All five agents from the group of DPP-4 inhibitors were marketed in the Czech Republic, Serbia, and Spain, whereas only four substances from the group of SGLT-2 inhibitors were found available in the Czech Republic, Poland, and Spain.

Less than 10% of all observations for authorized medicines had a retail price, except for Serbia (91.0%), Spain (46.3%), and France (17.3%). These discrepancies might be attributed to the wide range of authorized presentation forms, with many of them not being commercialized. The list of authorized medicines in Serbia included fewer presentation forms, which could explain the high percentages observed in this country. Serbia had fewer approved medicines than other countries, but over 90% of them had a calculated retail price, and thus likely to be available on the market. A number of studies have shown that the drivers responsible for differences between authorized and marketed medicines across countries are multifactorial. The European Federation of Pharmaceutical Industries and Associations (EFPIA) has highlighted that factors such as the authorization process, national pricing and reimbursement policies, market dynamics, and healthcare system characteristics can contribute to delays between obtaining marketing authorization and the application for pricing and reimbursement. These delays can significantly impact the availability of medicines. Significant differences in the mean time to reimbursement for innovative products were observed in Europe (2018–2021), from 128 days in Germany to 507 days in the Czech Republic, 508 days in France, followed by Spain (629 days), Bulgaria (705 days), Serbia (811 days), Poland (827 days), and Romania (918 days) [28]. Under the current EU law [18], it is not mandatory for a MAH to submit a pricing and reimbursement application after obtaining a marketing authorization. In some cases, patients do not have access to medicines even after they are included in the price catalogs and on the positive reimbursement lists. Sales data from 2015–2022 (published by IQVIA) illustrated that 18% of available medicines were not used in France (no registered sales), followed by Spain (22%), the Czech Republic (23%), Romania (24%), Poland (29%), and Bulgaria (32%) [28]. Many published studies showed differences in pharmaceutical price regulations across countries [29,30].

Over the last decade, a number of new antidiabetic agents were approved by EMA through the centralized procedure (marketing authorizations valid directly throughout all EU member states) and introduced into therapy: 5 DPP-4 inhibitors (sitagliptin and vildagliptin-2007, saxagliptin-2009, linagliptin-2011, alogliptin-2013), 6 GLP-1 receptor agonists (exenatide-2006, liraglutide-2009, lixisenatide-2013, dulaglutide and albiglutide-2014, semaglutide-2018), and 5 SGLT-2 inhibitors (dapagliflozin-2012, canagliflozin-2013, empagliflozin-2014, ertugliflozin-2018, sotagliflozin-2019) [31,32]. All studied countries had authorized products based on 5 INNs from the GLP-1 analogs group, but only Bulgaria, France, and Romania reported retail prices for all approved substances. Patients in the Czech Republic, Romania, Serbia, and Spain have wider access to semaglutide, as it is available in both injectable (subcutaneous) and pill (oral) forms. In other countries, semaglutide is only available as an injection. Semaglutide is the first GLP-1 agonist formulated as a tablet with a single daily administration. A systematic review based on twelve clinical trials concluded that oral semaglutide (14 mg) had better results in reducing HbA1c and body weight compared with placebo and other agents from the same class (exenatide, liraglutide, dulaglutide), and was almost as effective as its subcutaneous form [33]. All five DPP 4 inhibitors were available in the Czech Republic, Serbia, and Spain. Products containing alogliptine were unauthorized in Bulgaria and Romania and did not have an approved price in France and Poland. Similarly, linagliptine had no available price in France and Romania. France had the highest number of available brand names for medicines based on sitagliptine (12) and vildagliptine (8). Medicines with dapagliflozin and empagliflozin as active substances were available in all included countries; ertugliflozin was only marketed in the Czech Republic, Poland, and Spain, while sotagliflozin was not marketed in any of the included countries. Sotagliflozin was initially approved in the EU for adults with type 1 diabetes and overweight, and who struggle to achieve glycaemic control [34], but it was withdrawn from use in March 2022 at the request of the MAH for commercial reasons [35].

ADA (American Diabetes Association) guidelines from 2023 divide pharmacologic treatment based on therapeutic goals, comorbidities, and risk factors. Metformin or other substances used in monotherapy, but also combination therapy should be considered due to their efficacy in achieving therapeutic outcomes. When comorbidities or risk factors are present, the following recommended glucose-lowering agents are recommended: GLP-1 analogs and SGLT2 inhibitors (atherosclerotic cardiovascular disease and/or high-risk indicators); SGLT2 inhibitors (heart failure) or, if not tolerated, GLP-1 analogs (chronic kidney disease). If the goal is glucose-lowering and control, recommendations are made based on efficacy: GLP-1 RA (semaglutide, dulaglutide), tirzepatide, insulin, oral or injectable combinations (very high efficacy), metformin, other GLP-1 RA, SGLT2i, sulfonylurea, TZD (high), and DPP-4i (intermediate efficacy) [36].

Comparing the approved INNs grouped by class, our study showed that metformin, acarbose, and pioglitazone were both authorized and had a price in all countries (except for acarbose in Serbia and pioglitazone in France). Gliclazide and glimepiride were the only sulfonylureas found in medicines with an available retail price in all countries. The largest number of authorized active substances were in the Czech Republic and Poland (41), while Spain had the highest number of marketed substances (38). In Bulgaria, all authorized substances were found in commercialized medicines, and this situation is mirrored in Serbia (96%) and Spain (97%), where a similarly high percentage of authorized substances have price listings at pharmacies. It is important to have a diverse range of therapeutic options authorized, but they must also be available (having a price and no supply problems), accessible (affordable with respect to cost), and recommended for treatment to patients. Due to limited national regulatory and administrative reimbursement policies, not all medicines dispensed on a medical prescription are reimbursed by health insurers, and in such cases, the entire cost is to be paid by patients. For instance, this is the case for antidiabetic therapy in Poland. The Polish diabetes guidelines from 2022 mentioned the limited reimbursement of the new antidiabetic agents. According to the guidelines, six drug classes are recommended for type 2 diabetes treatment (leaving aside basal insulin): biguanides (metformin), sulfonylurea derivatives, SGLT-2 inhibitors, DPP-4 inhibitors, GLP-1 receptor agonists, or PPAR-γ agonists (pioglitazone) [37]. In line with international guidelines, diabetes care in Poland has the reduction of associated acute and chronic complications as its main therapeutic objective [38]. However, our study showed that there was a reduced number of marketed brand names compared with the approved ones, including several new antihyperglycemic agents with cardio-renal benefits (GLP-1 RA, SGLT-2 i, DPP-4 i). A study based on data from the National Health Funds (2012–2015) revealed that medicines with alpha-glucosidase inhibitors (volume-1%), thiazolidinediones (no sale), and DPP-4i (volume from 0.003% to 0.009%) were rarely prescribed in Poland [39].

Concerning brand names, more than half of the approved drugs had a pharmacy price in the majority of the studied countries. In Bulgaria, more than 70% of authorized commercial names, concentrations, and pharmaceutical forms had a retail price.

High numbers of marketed products containing metformin were observed, from seven brand names in Serbia to eighteen in Spain. This was expected since metformin has been for a long time the first-line agent for type 2 diabetes [40] and has been on the list of essential drugs of the World Health Organization (WHO) since 2011 [41]. Innovator names having a pharmacy price were common in most countries, with a few exceptions due to the existence of different names (for the same pharmaceutical form) depending on the country: metformin (Spain), glipizide, gliclazide (France, Spain), glimepiride (France), sitagliptin, vildagliptin, and sitagliptin + metformin. No generic common name was observed for sitagliptin in all the included countries. Generic medicines were absent for some antidiabetic agents, such as sitagliptin (Bulgaria), acarbose (Romania), and some fixed-dose combinations (except for metformin + vildagliptin in Bulgaria, France, and Romania). Repaglinide was found only as a generic in Bulgaria, Romania, and Serbia, and the combination metformin + vildagliptin was available only as a generic in Serbia. Pioglitazone was found only as a generic in Bulgaria, Poland, Romania, and Serbia.

It is well known that diabetes is a complex disease, and geriatric diabetic patients usually have other associated chronic conditions. Recent diabetes international guidelines and published studies provide recommendations for personalized treatment decisions, based not only on achieving optimal glycemic control, but also on patient lifestyle, personal preferences, and perception of the proposed therapy. Poor adherence to the treatment can result in treatment failure and an increased risk for complications [42]. A study conducted in Poland (DIABCON) indicated that only 65.1% of patients were compliant with the self-monitoring of blood glucose [43]. A retrospective study performed on elderly Spanish patients (≥65 years) revealed a 72% adherence to antidiabetic medicines [44].

Our study analyzed the approved and commercialized antidiabetic medicines in terms of pharmaceutical formulations and strengths. A reduced number of presentation forms can be a barrier to an individualized treatment. Eight authorized pharmaceutical forms were found in France, five in Bulgaria, and seven in the rest of the countries. At the INN level, all approved formulations were marketed only in Bulgaria. Glibenclamide was authorized as suspension and oral solid formulations in France and Spain, but only the solid form was a commercialized study. Metformin was approved and marketed only as filmed-coated tablets in Spain; therefore, a more personalized treatment was harder to implement. A study based on a randomized clinical trial has shown that extended-release formulations have better outcomes in terms of glycemic control, lipid, and certain inflammatory markers levels [45]. Furthermore, in a systematic review study, better adherence to treatment for extended-release metformin formulations was reported [46].

In the last few years, drug shortages have become a frequent and worrying problem across the world. Supply problems can cause distress for patients, increased time to find the medicine in the pharmacy or to obtain a modified prescription from the physician, as well as more work and pressure for healthcare professionals, especially pharmacists [47]. Usually, a therapeutic substitute for non-biological medicines is recommended if there are more commercial names. If not, another option can be to use a second- or third-line therapy, but this may increase the risk of having a sub-optimal glycemic level. Our analysis of the number of marketed brand names and strengths per active substance showed that only one product (one brand name with one strength) was available for certain sulfonylureas (glibenclamide—Romania, Serbia, Spain; glipizide—the Czech Republic, Poland, Romania, Spain; glisentide—Spain); acarbose (the Czech Republic) and DPP-4i (sitagliptin—Bulgaria, Poland; vildagliptin— the Czech Republic, Poland; saxagliptin— the Czech Republic, France, Romania, Serbia; alogliptin—Serbia; and linagliptin—all countries that have authorized products). Concerning GLP-1 agonists, only one pharmaceutical product was marketed for exenatide (Bulgaria), liraglutide (Bulgaria, the Czech Republic, Serbia, Spain), and lixisenatide (Romania). Consequently, in case of insufficient supply, the absence of these pharmaceutical products can affect patient treatment, leaving health professionals as pharmacists without therapeutic alternatives. Patients must return to their physicians in order to receive another medicine (from the same class or a distinct one), facing anxiety, wasted time, and possible adherence problems due to the treatment change. A survey study addressed to the German community and hospital pharmacists in 2016 revealed negative consequences for patient care due to drug shortages, such as medication errors and less appropriate medicine or dosage forms selected for administration [48]. A diverse range of pharmacological options can ensure a suitable, continuous medical regime.

Most countries rely on imports, which are vulnerable to drug shortages in case of supply problems. Such issues were aggravated by the COVID-19 pandemic, which highlighted the dependence of manufacturers on external sources of excipients, active pharmaceutical ingredients (API), and packaging materials [49]. Through a resolution adopted in September 2021, the European Union Parliament has urged European manufacturers to be more self-reliant and to develop a stronger local production of substances in order to prevent future health crises. A few years ago, in the United States, 63% and 80%, respectively, of the manufacturing sites for APIs and finished products were outside the country. Additionally, there was a gap between the total number of authorized products and those with a retail price. Only 39% of generic-approved medicines were available in June 2019 [50]. In our study, local companies (created in the studied countries) were included in the top 10, including, for instance, Biogaran [51], Arrow Generiques [52], EG Labo-Laboratoires EuroGenerics [53] (France), Polpharma Zakłady Farmaceutyczne (Poland) [54], Arena Group (Romania) [55], Zentiva (the Czech Republic) [56], and Galenika (Serbia) [57]. Sales reports from the literature indicate that the key players in the diabetes market are Eli Lilly, Novo Nordisk, AstraZeneca, Sanofi, Merck & Co, Novartis, and Boehringer Ingelheim [58], and our results are consistent with these findings.

In the included countries of this study with lower values for marketed pharmaceutical forms compared with the authorized ones, clinicians might find it challenging to create individualized therapeutic interventions. This can lead to reduced compliance and non-optimal glycaemic control, with a higher risk of developing associated complications and increased costs for health systems. An analysis of the estimated total cost for preventable hospitalizations for diabetic patients in the United States showed an annual increase in the costs of long-term complications from USD 2.7 billion in 2001 (59% of the total) to USD 3.3 billion in 2014 (57% of total) [59]. Moreover, a study conducted in China showed that the direct medical cost for diabetic patients increased directly with the number of complications for the study period (2009–2011) [60].

Study limitations. The current study took into consideration data from national lists of medicines with pharmacy prices. However, not all medicines with a price can be found in the pharmacy due to various medicines shortages; it is expected that the number of marketed medicines in each country of study is actually slightly smaller than the one of medicines with a price.

## 4. Materials and Methods

### 4.1. Data Source

This study was conducted from September 2021 to April 2022 and encompassed a comparative analysis of seven countries: Romania (R), Serbia (SB), Spain (SP), Bulgaria (B), Poland (P), France (F), and the Czech Republic (CR). Information about authorized medicines and those with a pharmacy (retail) price was acquired through primary research conducted on databases from the official websites of the national authorities of the included countries [61,62,63,64,65,66,67,68,69,70,71,72,73] or through experts from participating countries.

### 4.2. Data Collection and National Context

In this study, we included medicines classified within the ATC (Anatomical Therapeutic Chemical Classification System) group A10B (blood glucose-lowering drugs, excluding insulin). Data from the ATC/DDD (defined daily dose) index was used to identify the therapeutic classes corresponding to antidiabetic drugs [74].

The ATC codes for the therapeutic groups used in this study were the following:

- A10BA (biguanides);
- A10BB (sulfonylurea);
- A10BD (oral fixed combinations);
- A10F (alpha-glucosidase inhibitors);
- A10BG (thiazolidinediones, TDZ);
- A10BH (dipeptidyl peptidase 4 (DPP-4) inhibitors, DPP-4i);
- A10J (glucagon-like peptide-1 (GLP-1) analogues, GLP-1 receptor agonists, GLP-1 RA);
- A10K (sodium-glucose co-transporter 2 (SGLT2) inhibitors);
- A10X (other blood glucose-lowering drugs, excluding insulins).

In each country, medicines for diabetes are dispensed based on a medical prescription, and the cost of treatment can usually be refunded at a certain level by the (public) health insurance funds. 

Medicinal products having their marketing authorization revoked (lacking marketing authorization) after data collection were excluded from the analysis. In the majority of the included countries, the list of authorized medicines contained several forms of presentation, mentioned in the summary of product characteristics. However, the data from CIMA (Medicine Online Information Center of AEMPS—The Spanish Agency for Medicines and Health Products) did not include all authorized presentations [66]. Consequently, summaries of product characteristics were analyzed in order to collect all the authorized types of packaging and to add them to the list of authorized medicines.

Serbia uses an officially published list of maximum wholesale price levels for prescription medicines, so pharmacy prices are not officially published. Therefore, in this study, pharmacy prices were calculated based on wholesale published prices [72]. In the other included countries, pharmacy price information was obtained directly from the official websites of the national authorities [64,68,69,70,71,73].

### 4.3. Data Analysis

To analyze the national data about authorized medicines in comparison with medicines having a pharmacy (retail) price, the following attributes were selected: ATC code and class, INN (International Non-proprietary Name), MAH (Marketing Authorization Holder), brand name, pharmaceutical form, strength and status of medicine (innovative, generic). 

The term “observation” within a data set in this study refers to medicine having a certain brand name, strength, pharmaceutical form, and packaging (a presentation form). Many observations can be available in the national lists of approved medicines for a certain brand name, each corresponding to different characteristics (strengths, pharmaceutical forms, packaging). In this study, the term “marketed” or “commercialized” refers to a medicine that has a market authorization and a price, so that it may be found in pharmacies. Moreover, the term “available” or “availability” is also used for medicines with a pharmacy price. However, the existence of a medication price does not necessarily confirm its physical availability, as in a minority of situations supply chain issues might affect its accessibility.

We analyzed the data by active substance, and then compiled and organized the results by therapeutic group (expressed as ATC code). We reported the number of INNs (absolute values) by therapeutic group for each country (authorized medicines and those with a price) and compared it with the total number of authorized active substances by class in the seven countries included in this study. For the other characteristics, data were reported individually, in relation to the situation of each country.

Data were analyzed in order to identify possible medicines that may be at risk in case of supply problems, for instance, products having only one brand name with a reduced number of presentation forms. Therefore, the number of brand names, strengths, innovators, and generic names were analyzed per active substance for marketed medicines and then presented by class, in relation to the value “1”. Counts were used for brand names and pharmaceutical forms analyzed by ATC group. General attributes such as the count of observations (presentation forms) of medicines having a retail price were compared (as percentages out of the total number of authorized presentation forms) with those of all corresponding authorized medicines in each country.

The percentages of different pharmaceutical forms were calculated for each category and strength separately for authorized and marketed medicines, out of the total number of authorized and marketed strengths, respectively.

We considered as distinct names (products) medicines that had a similar name, with the same active substance and MAH, but with a different pharmaceutical form (Glucophage—filmed tablet vs. Glucophage XR—prolonged-release tablet; Avamina-filmed tablet vs. Avamina SR—prolonged-release tablet).

We considered various linguistic versions of the same INN as the same name (e.g., “metformin”, “metformine”, “metformina”; “akarbosa”, “akarboza”, “acarbose”) or names with a difference generated by the pharmaceutical form: Siofor prolong (the Czech Republic), Siofor SR (Bulgaria), and Siofor XR (Poland). However, we considered different brand names for innovator medicines distinguished by one or several letters such as “Amaryl” (Bulgaria, the Czech Republic, Poland, Romania, Serbia, Spain) versus “Amarel” (France), or “Minodiab” (Spain) versus “Minidiab” (the Czech Republic).

An analysis of the active substances formulated as approved medicines and those products with pharmacy prices is shown by the country in the electronic Supplementary Materials (Table S1). Furthermore, the number of commercial names by active substance (all medicines and those with a price) (Table S2), details about common commercial names encountered in ≥2 countries (countries, number of medicine names and innovator or generic status) (Tables S3 and S4), number of innovator and generic medicines (Tables S5 and S6), types of pharmaceutical forms and number of strengths by INN (Tables S7 and S8) were also analyzed. A top ten of MAHs was also provided in the electronic Supplementary Materials (Tables S9 and S10), selected by the number of INNs and brand names; in the case of the same values, the number of observations and strengths were also taken into consideration.

## 5. Conclusions

This study provides a comprehensive view of the range of authorized antidiabetic agents (excluding insulin) available for patients’ treatment across seven European countries. Not all authorized drugs have a pharmacy price or are reimbursed by national health insurance funds. Awareness of the full spectrum of pharmacological options is important in ensuring equitable patient access to treatment and achieving a good clinical outcome, taking rapid measures in health crises, and making well-informed decisions based on clinical evidence.

## Figures and Tables

**Table 1:** Characteristics of the range of authorized medicines having a pharmacy price (absolute values and percentages), compared with the authorized medicines at a national level.

Country	Number of Observations (%)	Number of Brand Names (%)	Number of INNs * (%)	Number of Strengths (%)	Number of Pharmaceutical Forms (%)	Number of MAHs ** (%)
Bulgaria	115 (6.2%)	56 (71.8%)	27 (100.0%)	34 (87.2%)	5 (100.0%)	25 (73.5%)
Czech Republic	235 (7.5%)	70 (49.6%)	33 (80.5%)	46 (67.6%)	6 (85.7%)	29 (69.0%)
France	439 (17.3%)	116 (60.4%)	22 (59.5%)	34 (55.7%)	5 (62.5%)	30 (62.5%)
Poland	211 (8.9%)	46 (26.0%)	15 (36.6%)	27 (38.6%)	5 (71.4%)	30 (50.0%)
Romania	115 (8.9%)	49 (58.3%)	27 (77.1%)	44 (77.2%)	7 (100.0%)	21 (65.6%)
Serbia ***	193 (91.0%)	49 (90.7%)	24 (96.0%)	41 (100.0%)	5 (100.0%)	23 (95.8%)
Spain	320 (46.3%)	123 (57.7%)	38 (97.4%)	51 (86.4%)	6 (85.7%)	45 (70.3%)

* INN = International Non-proprietary Name; ** MAH = marketing authorization holder; *** pharmacy price = pharmacy calculated price; in Serbia, there is no officially published list with pharmacy prices, but pharmacy prices can be calculated from the wholesale price level that is published.

**Table 2:** Number of brand names per INN for medicines with a pharmacy price by therapeutic group and country.

Therapeutic Group	Number of Brand Names/INN						
(ATC Code)	Bulgaria	Czech Republic	France	Poland	Romania	Serbia	Spain
Biguanides (A10BA)	>1	>1	>1	>1	>1	>1	>1
Sulfonylureas (A10BB)	>1	>1	>1	>1	≥1	>1	>1
Combinations (A10BD)	≥1	≥1	>1	N/A	≥1	≥1	≥1
α-glucosidase inhibitors (A10BF)	>1	1	>1	>1	1	N/A	>1
Thiazolidinediones (A10BG)	>1	>1	N/A	1	1	1	>1
DPP-4i (A10BH)	≥1	≥1	>1	1	>1	≥1	>1
GLP-1 RA (A10BJ)	1	≥1	≥1	1	≥1	≥1	≥1
SGLT2i (A10BK)	1	1	1	1	1	1	1
Other blood glucose-lowering drugs,	>1	>1	>1	N/A	>1	>1	≥1
excluding insulin (A10X)							

N/A = not available, there is no medicine with pharmacy price based on oral fixed combinations in Poland.

**Table 3:** Number of innovator medicines names per INN for all medicines with marketing authorization by therapeutic group and country.

Therapeutic Group	Innovator Medicine Name/INN							Generic Medicine Name/INN						
(ATC Code)	B	CZ	F	P	R	SB	SP	B	CZ	F	P	R	SB	SP
Biguanides (A10BA)	>1	>1	1	>1	>1	>1	1	>1	>1	>1	>1	>1	>1	>1
Sulfonylureas (A10BB)	1	1	≥1	≥1	≤1	1	≥1	>1	>1 *	≥1	≥1 *	≥1 *	>1 *	<1 **
Combinations (A10BD)	≤1	≥1	≥1	≥1	≥1	<1 **	≥1	≥1 *	>1 *	>1 *	>1 *	≥1 *	≤1 **	≤1 **
α-glucosidase inhibitors (A10BF)	1	N/A	1	1	1	1	1	1	>1	>1	>1	N/A	N/A	>1
Thiazolidinediones (A10BG)	N/A	1	1	>1	1	N/A	1	>1	>1	N/A	>1	>1	1	>1
DPP-4i (A10BH)	1	≥1	≥1	≥1	≥1	1	≥1	>1 *	>1 *	≥1 *	>1 *	>1 *	<1 **	>1 *
GLP-1 RA (A10BJ)	1	≥1	≥1	≥1	≥1	≥1	≥1	N/A	N/A	N/A	N/A	N/A	N/A	N/A
SGLT2i (A10BK)	1	≥1	1	≥1	1	1	≥1	N/A	N/A	N/A	N/A	N/A	N/A	N/A
Other blood glucose-lowering drugs,	N/A	≥1	1	≥1	≤1	N/A	≥1	>1	>1 *	>1	>1	>1 *	>1	<1 **
excluding insulin (A10X)														

N/A = innovator/generic medicine not available; * for some INNs, there is no innovator/generic medicine with a marketing authorization; ** for some INNs, there is more than one innovator/generic medicine.

**Table 4:** Number of innovator and generic medicines names per INN for medicines having a pharmacy price by therapeutic group and country.

Therapeutic Group	Innovator Medicine Name/INN							Generic Medicine Name/INN						
(ATC Code)	B	CZ	F	P	R	SB	SP	B	CZ	F	P	R	SB	SP
Biguanides (A10BA)	>1	>1	1	>1	>1	>1	1	>1	>1	>1	>1	>1	>1	>1
Sulfonylureas (A10BB)	1	1	1	≤1	≤1	1	1	>1	>1 *	>1 *	≥1 *	≥1 *	≤1 **	>1 **
Combinations (A10BD)	≤1	≥1	≥1	N/A	≥1	≤1	≥1	≤1 **	N/A	<1 **	N/A	≥1 *	≤1 **	≤1 **
α-glucosidase inhibitors (A10BF)	1	N/A	1	1	1	N/A	1	1	1	>1	1	N/A	N/A	>1
Thiazolidinediones (A10BG)	N/A	1	N/A	N/A	N/A	N/A	1	>1	>1	N/A	1	1	1	>1
DPP-4i (A10BH)	1	≥1	≥1	≤1	1	≤1	≥1	≤1	N/A	>1 *	≤1	<1 **	≤1 **	N/A
GLP-1 RA (A10BJ)	1	≥1	>1	1	≥1	≥1	≥1	N/A	N/A	N/A	N/A	N/A	N/A	N/A
SGLT2i (A10BK)	1	≥1	1	1	1	1	≥1	N/A	N/A	N/A	N/A	N/A	N/A	N/A
Other blood glucose-lowering drugs,	N/A	1	1	N/A	N/A	N/A	≥1	>1	>1	>1	N/A	>1	>1	<1 **
excluding insulin (A10X)														

N/A = innovator/generic medicine not available; * for some INNs, there is no innovator/generic medicine with a marketing authorization; ** for some INNs, there is more than one innovator/generic medicine.

**Table 5:** Number of strengths per INN for medicines with a pharmacy price by therapeutic group across the studied countries.

Therapeutic Group	Number of Strengths/INN						
(ATC Code)	Bulgaria	Czech Republic	France	Poland	Romania	Serbia	Spain
Biguanides (A10BA)	>1	>1	>1	>1	>1	>1	>1
Sulfonylureas (A10BB)	>1	≥1	>1	≥1	≥1	≥1	≥1
Combinations (A10BD)	≥1	≥1	≥1	N/A	≥1	≥1	≥1
α-glucosidase inhibitors (A10BF)	1	1	>1	>1	>1	N/A	>1
Thiazolidinediones (A10BG)	>1	>1	N/A	>1	>1	>1	>1
DPP-4i (A10BH)	1	≥1	≥1	1	≥1	≥1	≥1
GLP-1 RA (A10J)	≥1	≥1	≥1	>1	≥1	≥1	≥1
SGLT2i (A10K)	1	≥1	≥1	≥1	≥1	>1	≥1
Other blood glucose-lowering drugs, excluding insulins (A10X)	>1	>1	>1	N/A	>1	>1	>1

N/A = medicine not available.

## Data Availability

The original contributions presented in this study are included in the article/Supplementary Materials. Further inquiries can be directed to the corresponding author.

## Supplementary Materials

The following supporting information can be downloaded at: https://www.mdpi.com/article/10.3390/ph17060793/s1, Table S1: active substances found in antidiabetic medicines with a marketing authorization (N) and a retail price (n) for each country of study; Table S2: number of brand names for all authorized medicines compared with medicines having a pharmacy price by ATC code, INN and country; Table S3: overview of the common brand names (all approved medicines) in the countries of study; Table S4: overview of the common brand names (having a pharmacy price) in the countries of study; Table S5: number of innovator and generic brand names per INN for all authorized medicines; Table S6: number of innovator and generic brand names per INN for medicines with pharmacy price; Table S7: types of pharmaceutical forms; Table S8: number of strengths by INN and country for all authorized and marketed medicines; Table S9: top 10 marketing authorization holders for all authorized medicines; Table S10: top 10 marketing authorization holders for marketed medicines.

## References

[1] Cousin E., Duncan B.B., Stein C., Ong K.L., Vos T., Abbafati C., Haque S. (2022). Diabetes mortality and trends before 25 years of age: An analysis of the Global Burden of Disease Study 2019. Lancet Diabetes Endocrinol..

[2] Liang D., Cai X., Guan Q., Ou Y., Zheng X., Lin X. (2023). Burden of type 1 and type 2 diabetes and high fasting plasma glucose in Europe, 1990–2019: A comprehensive analysis from the global burden of disease study 2019. Front. Endocrinol..

[3] Cannon A., Handelsman Y., Heile M., Shannon M. (2018). Burden of Illness in Type 2 Diabetes Mellitus. J. Manag. Care Spec. Pharm..

[4] Yang X., Sun J., Zhang W. (2024). Global trends in burden of type 2 diabetes attributable to physical inactivity across 204 countries and territories, 1990–2019. Front. Endocrinol..

[5] International Diabetes Federation (2021). IDF Diabetes Atlas.

[6] Lombardo F., Maggini M., Gruden G., Bruno G. (2013). Temporal Trend in Hospitalizations for Acute Diabetic Complications: A Nationwide Study, Italy, 2001–2010. PLoS ONE.

[7] Stratton I.M., Adler A.I., Neil H.A., Matthews D.R., Manley S.E., Cull C.A., Hadden D., Turner R.C., Holman R.R. (2000). Association of glycaemia with macrovascular and microvascular complications of type 2 diabetes (UKPDS 35): Prospective observational study. BMJ.

[8] Seuring T., Archangelidi O., Suhrcke M. (2015). The Economic Costs of Type 2 Diabetes: A Global Systematic Review. PharmacoEconomics.

[9] Asmat K., Dhamani K., Gul R., Froelicher E.S. (2022). The effectiveness of patient-centered care vs. Usual care in type 2 diabetes self-management: A systematic review and meta-analysis. Front. Public. Health.

[10] Blonde L., Umpierrez G.E., Reddy S.S., McGill J.B., Berga S.L., Bush M., Chandrasekaran S., DeFronzo R.A., Einhorn D., Galindo R.J. (2022). American Association of Clinical Endocrinology Clinical Practice Guideline: Developing a Diabetes Mellitus Comprehensive Care Plan-2022 Update. Endocr. Pract..

[11] Menditto E., Orlando V., De Rosa G., Minghetti P., Musazzi U.M., Cahir C., Kurczewska-Michalak M., Kardas P., Costa E., Sousa Lobo J.M. (2020). Patient Centric Pharmaceutical Drug Product Design—The Impact on Medication Adherence. Pharmaceutics.

[12] Inzucchi S.E., Bergenstal R.M., Buse J.B., Diamant M., Ferrannini E., Nauck M., Peters A.L., Tsapas A., Wender R., Matthews D.R. (2014). Management of Hyperglycemia in Type 2 Diabetes, 2015: A Patient-Centered Approach: Update to a Position Statement of the American Diabetes Association and the European Association for the Study of Diabetes. Diabetes Care.

[13] World Health Organization (2006). Quality of Care: A Process for Making Strategic Choices in Health Systems.

[14] Medicine Agency Marketing Authorisation. https://www.ema.europa.eu/en/human-regulatory/marketing-authorisation.

[15] (2004). Regulation (EC) No 726/2004 of the European Parliament and of the Council of 31 March 2004 Laying down Union Procedures for the Authorisation and Supervision of Medicinal Products for Human Use and Establishing a European Medicines Agency. https://eur-lex.europa.eu/eli/reg/2004/726/oj.

[16] Bade C., Olsacher A., Boehme P., Truebel H., Fehring L. (2023). Reasons for supply side driven drug shortages—A mixed-methods study on first-level, higher-level, and root causes from the perspective of marketing authorization holders. Res. Soc. Adm. Pharm..

[17] (2022). HMA/EMA Task Force on Availability of Authorised Medicines for Human and Veterinary Use (TFAAM) Terms of Reference. https://www.hma.eu/fileadmin/dateien/HMA_joint/00-_About_HMA/03-Working_Groups/TF_Availability/2022_09_HMA-EMA_TF_Availability_ToR.pdf.

[18] European Commission A Pharmaceutical Strategy for Europe. https://ec.europa.eu/health/human-use/strategy_en..

[19] (2021). European Commission. Introducing HERA, the European Health Emergency Preparedness and Response Authority, the Next Step towards Completing the European Health Union, Brussel.

[20] (2022). Regulation (EU) 2022/123 of the European Parliament and of the Council of 25 January 2022 on a Reinforced Role for the European Medicines Agency in Crisis Preparedness and Management for Medicinal Products and Medical Devices. https://eur-lex.europa.eu/legal-content/EN/TXT/?uri=CELEX:32022R0123.

[21] European Commission Reform of the EU Pharmaceutical Legislation. https://health.ec.europa.eu/medicinal-products/pharmaceutical-strategy-europe/reform-eu-pharmaceutical-legislation_en.

[22] Overbeek J.A., Heintjes E.M., Prieto-Alhambra D., Blin P., Lassalle R., Hall G.C., Lapi F., Bianchini E., Hammar N., Bezemer I.D. (2017). Type 2 Diabetes Mellitus Treatment Patterns Across Europe: A Population-based Multi-database Study. Clin. Ther..

[23] Pantalone K.M., Hobbs T.M., Wells B.J., Kong S.X., Kattan M.W., Bouchard J., Yu C., Sakurada B., Milinovich A., Weng W. (2015). Clinical characteristics, complications, comorbidities and treatment patterns among patients with type 2 diabetes mellitus in a large integrated health system. BMJ Open Diabetes Res. Care.

[24] Nishimura R., Kato H., Kisanuki K., Oh A., Hiroi S., Onishi Y., Guelfucci F., Shimasaki Y. (2019). Treatment patterns, persistence and adherence rates in patients with type 2 diabetes mellitus in Japan: A claims-based cohort study. BMJ Open.

[25] Moura A.M., Martins S.O., Raposo J. (2021). Consumption of antidiabetic medicines in Portugal: Results of a temporal data analysis of a thirteen-year study (2005–2017). BMC Endocr. Disord..

[26] Gimeno J.A., Cánovas G., Durán A. (2021). Factors Associated with Adherence to Clinical Practice Guidelines for Patients with Type 2 Diabetes Mellitus: Results of a Spanish Delphi Consensus. J. Diabetes Res..

[27] Mardetko N., Nabergoj Makovec U., Locatelli I., Janez A., Kos M. (2021). Uptake of new antidiabetic medicines in 11 European countries. BMC Endocr. Disord..

[28] (2023). The European Federation of Pharmaceutical Industries and Associations (EFPIA) and CRA, The Root Cause of Unavailability and Delay to Innovative Medicines. https://www.efpia.eu/media/677292/cra-efpia-root-causes-unavailability-delay-080423-final.pdf.

[29] Leopold C., Mantel-Teeuwisse A.K., Vogler S., de Joncheere K., Laing R.O., Leufkens H.G.M. (2013). Is Europe still heading to a common price level for on-patent medicines? An exploratory study among 15 Western European countries. Health Policy.

[30] Petrou P., Vandoros S. (2016). Pharmaceutical price comparisons across the European Union and relative affordability in Cyprus. Health Policy Technol..

[31] Blind E., Janssen H., Dunder K., de Graeff P.A. (2018). The European Medicines Agency’s approval of new medicines for type 2 diabetes. Diabetes Obes. Metab..

[32] EMA, Zynquista. https://www.ema.europa.eu/en/medicines/human/EPAR/zynquista.

[33] Alhindi Y., Avery A. (2022). The efficacy and safety of oral semaglutide for glycaemic management in adults with type 2 diabetes compared to subcutaneous semaglutide, placebo, and other GLP-1 RA comparators: A systematic review and network meta-analysis. Contemp. Clin. Trials Commun..

[34] Markham A., Keam S.J. (2019). Sotagliflozin: First Global Approval. Drugs.

[35] EMA, Public statement, Zynquista Withdrawal of the marketing authorisation in the European Union, 19 May 2022. https://www.ema.europa.eu/en/documents/public-statement/public-statement-zynquista-withdrawal-marketing-authorisation-european-union_en.pdf.

[36] American Diabetes Association (2023). Standards of Care in Diabetes—2023. Diabetes Care.

[37] Araszkiewicz A., Bandurska-Stankiewicz E., Borys S., Budzyński A., Cyganek K., Cypryk K., Czech A., Czupryniak L., Drzewoski J., Dzida G. (2022). Guidelines on the management of patients with diabetes. A position of Diabetes Poland. Curr. Top. Diabetes.

[38] Malecki M.T., Klupa T., Bociąga-Jasik M., Kowalska I., Gumprecht J., Strojek K., Zozulińska-Ziółkiewicz D., Czupryniak L., Hohendorff J., Kania M. (2022). Guidelines of the Diabetes Poland on the therapeutic management and glycemic monitoring in diabetic patients in the COVID-19 pandemic and other viral pandemics. Curr. Top. Diabetes.

[39] Śliwczyński A., Brzozowska M., Jacyna A., Iltchev P., Iwańczuk T., Wierzba W., Marczak M., Orlewska K., Szymański P., Orlewska E. (2017). Drug-class-specific changes in the volume and cost of antidiabetic medications in Poland between 2012 and 2015. PLoS ONE.

[40] Bouchi R., Sugiyama T., Goto A., Imai K., Ihana-Sugiyama N., Ohsugi M., Yamauchi T., Kadowaki T., Ueki K. (2022). Retrospective nationwide study on the trends in first-line antidiabetic medication for patients with type 2 diabetes in Japan. J. Diabetes Investig..

[41] Grytsai O., Myrgorodska I., Rocchi S., Ronco C., Benhida R. (2021). Biguanides drugs: Past success stories and promising future for drug discovery. Eur. J. Med. Chem..

[42] Piragine E., Petri D., Martelli A., Calderone V., Lucenteforte E. (2023). Adherence to Oral Antidiabetic Drugs in Patients with Type 2 Diabetes: Systematic Review and Meta-Analysis. J. Clin. Med..

[43] Rokicka D., Wróbel M., Szymborska-Kajanek A., Bożek A., Strojek K. (2018). Assessment of compliance to self-monitoring of blood glucose in type 2 diabetic patients and level of implementation of Polish Diabetes Association Recommendation for general practitioners. Clin. Diabetol..

[44] Juste A.M., Miguel A.G., Plou B.P., Rubio F.G., Pascual-Salcedo M.M.A., Menditto E., Torres A.P. (2019). Adherence to treatment of hypertension, hypercholesterolaemia and diabetes in an elderly population of a Spanish cohort. Med. Clin..

[45] Derosa G., D’Angelo A., Romano D., Maffioli P. (2017). Effects of metformin extended release compared to immediate release formula on glycemic control and glycemic variability in patients with type 2 diabetes. Drug Des. Devel Ther..

[46] Tan J., Wang Y., Liu S., Shi Q., Zhou X., Zhou Y., Yang X., Chen P., Li S. (2021). Long-Acting Metformin Vs. Metformin Immediate Release in Patients With Type 2 Diabetes: A Systematic Review. Front. Pharmacol..

[47] Walker J., Chaar B.B., Vera N., Pillai A.S., Lim J.S., Bero L., Moles R.J. (2017). Medicine shortages in Fiji: A qualitative exploration of stakeholders’ views. PLoS ONE.

[48] Said A., Goebel R., Ganso M., Zagermann-Muncke P., Schulz M. (2018). Drug shortages may compromise patient safety: Results of a survey of the reference pharmacies of the Drug Commission of German Pharmacists. Health Policy.

[49] Jongh T., Becker D., Boulestreau M., Davé A., Dijkstal F., King R., Petrosova L., Varnai P., Vis C., Spit W. (2021). Future-Proofing Pharmaceutical Legislation—Study on Medicine Shortages—Final Report (Revised).

[50] Federation Drug Agency (2019). Drug Shortages. Root Causes and Potential Solutions. https://www.fda.gov/media/131130/download?attachment.

[51] Biogaran. https://biogaran.com/en/.

[52] Laboratoire Arrow. https://www.laboratoire-arrow.com/.

[53] Laboratoire EG LABO. https://www.eglabo.fr/.

[54] Polpharma Zakłady Farmaceutyczne. https://polpharma.pl/.

[55] Arena Group. https://arenagroup.com/.

[56] Zentiva. https://www.zentiva.com/.

[57] Galenika. https://galenika.rs/.

[58] Market Research Future. https://www.marketresearchfuture.com/reports/diabetes-drug-market-1160.

[59] Shrestha S.S., Zhang P., Hora I., Geiss S.L., Luman T.E., Gregg W.E. (2019). Factors Contributing to Increases in Diabetes-Related Preventable Hospitalization Costs among U.S. Adults During 2001–2014. Diabetes Care.

[60] Huang Y., Vemer P., Zhu J., Postma M.J., Chen W. (2016). Economic Burden in Chinese Patients with Diabetes Mellitus Using Electronic Insurance Claims Data. PLoS ONE.

[61] National Agency for Medicines and Medical Devices of Romania Medicinal Product Index. https://nomenclator.anm.ro/medicamente.

[62] Bulgarian Drug Agency Register of Pharmaceutical Products. https://www.bda.bg/en/registers/register-of-pharmaceutical-products.

[63] Medicines and Medical Devices Agency of Serbia (Alims) Human Medicines. https://www.alims.gov.rs/english/medicinal-products/search-for-human-medicines/.

[64] State Institute for Drug Control Medicinal Product Database. https://www.sukl.cz/vyhledavani-v-databazi-leku.

[65] Office for Registration of Medicinal Products, Medical Devices and Biocidal Products Register of Medicinal Products. https://www.urpl.gov.pl/pl/produkty-lecznicze/zagadnienia-rejestracyjne/rejestr-produkt%C3%B3w-leczniczych.

[66] The Spanish Agency for Medicines and Health Products (AEMPS) AEMPS online drug information center—CIMA. https://cima.aemps.es/cima/publico/lista.html.

[67] The National Agency for the Safety of Medicines and Health Products (ANSM) Directory of Pharmaceutical Specialties. http://agence-prd.ansm.sante.fr/php/ecodex/index.php#result.

[68] The Ministry of Health and Prevention Public Drug Database. https://base-donnees-publique.medicaments.gouv.fr/index.php.

[69] The Ministry of Health Nomenclátor de Facturación. https://www.sanidad.gob.es/en/profesionales/nomenclator.do.

[70] National Council on Prices and Reimbursement of Medicinal Products. https://www.ncpr.bg/en/.

[71] Ministry of Health, National Public Catalog of Maximum Prices of Medicines for Human Use. https://ms.ro/en/ministry/structure/directia-politica-medicamentului-si-a-dispozitivelor-medicale/preturi-medicamente.

[72] Decision on the highest prices of drugs for use in human medicine, which are subject to prescription. Official Gazette of the Republic of Serbia No. 48/2021-8, 90/2021-3, 92/2021-12 (corr), 125/2021-42, 18/2022-65, 67/2022-75, 107/2022-8, 141/2022-178.

[73] Ministry of Health Annex to the announcement of the Minister of Health 2022, Poland. https://www.gov.pl/web/zdrowie/obwieszczenia-ministra-zdrowia-lista-lekow-refundowanych.

[74] WHO Collaborating Centre for Drug Statistics Methodology ATC/DDD Index. https://www.whocc.no/atc_ddd_index/.

