# Supplementary material for: Approved and Commercialized Antidiabetic Medicines (Excluding Insulin) in Seven European Countries—A Cross-Sectional Comparison
Source: Pharmaceuticals (Basel). 2024 Jun 17;17(6):793. doi: 10.3390/ph17060793 (PMC11207096; doi:10.3390/ph17060793)
Supplement: Supplementary file 1 [file pharmaceuticals-17-00793-s001.zip › pharmaceuticals-3040114-supplementary.pdf]

Supplementary Table S1. Active substances found in antidiabetic medicines with a marketing authorization (N) and a retail price (n) for each country of study

ATC code	INN	Bulgaria		Czech Republic		France		Poland		Romania		Serbia		Spain	
		**N**	**n**	**N**	**n**	**N**	**n**	**N**	**n**	**N**	**n**	**N**	**n**	**N**	**n**
A10BA02	Metforminum	x	x	x	x	x	x	x	x	x	x	x	x	x	x
A10BB01	Glibenclamidum	N/A	N/A	x	N/A	x	x	x	N/A	x	x	x	x	x	x
A10BB07	Glipizidum	N/A	N/A	x	x	x	x	x	x	x	x	N/A	N/A	x	x
A10BB08	Gliquidonum	N/A	N/A	x	x	N/A	N/A	x	N/A	x	x	N/A	N/A	N/A	N/A
A10BB09	Gliclazidum	x	x	x	x	x	x	x	x	x	x	x	x	x	x
A10BB12	Glimepiridum	x	x	x	x	x	x	x	x	x	x	x	x	x	x
A10BB91	Glisentidum	N/A	N/A	N/A	N/A	N/A	N/A	N/A	N/A	N/A	N/A	N/A	N/A	x	x
A10BD02	Metforminum + Glibenclamidum	N/A	N/A	x	x	x	x	N/A	N/A	x	x	N/A	N/A	N/A	N/A
A10BD05	Pioglitazonum + Metforminum	x	x	x	x	x	N/A	x	N/A	x	N/A	N/A	N/A	x	x
A10BD06	Pioglitazonum + Glimepiridum	N/A	N/A	x	N/A	x	N/A	x	N/A	N/A	N/A	N/A	N/A	x	x
A10BD07	Sitagliptinum + Metforminum	x	x	x	x	x	x	x	N/A	x	x	x	x	x	x
A10BD08	Vildagliptinum + Metforminum	x	x	x	x	x	x	x	N/A	x	x	x	x	x	x
A10BD09	Pioglitazonum + Alogliptinum	N/A	N/A	x	x	N/A	N/A	x	N/A	N/A	N/A	N/A	N/A	x	x
A10BD10	Saxagliptinum + Metforminum	x	x	x	x	x	x	x	N/A	x	x	x	x	x	x
A10BD11	Linagliptinum + Metforminum	x	x	x	x	x	N/A	x	N/A	x	N/A	N/A	N/A	x	x
A10BD13	Metforminum + Alogliptinum	N/A	N/A	x	x	x	N/A	x	N/A	N/A	N/A	N/A	N/A	x	x
A10BD15	Dapagliflozinum + Metforminum	x	x	x	x	x	x	x	N/A	x	x	x	x	x	x
A10BD16	Canagliflozinum + Metforminum	x	x	x	x	x	N/A	x	N/A	x	N/A	N/A	N/A	x	x
A10BD19	Linagliptinum + Empagliflozinum	x	x	x	N/A	x	N/A	x	N/A	N/A	N/A	N/A	N/A	x	x
A10BD20	Empagliflozinum+Metforminum	x	x	x	x	x	x	x	N/A	x	x	x	x	x	x
A10BD21	Saxagliptinum + Dapagliflozinum	x	x	x	N/A	x	N/A	x	N/A	x	x	x	x	x	N/A
A10BD23	Ertugliflozinum + Metforminum	N/A	N/A	x	x	x	N/A	x	N/A	x	N/A	N/A	N/A	x	x
A10BD24	Ertugliflozinum + Sitagliptinum	N/A	N/A	x	N/A	x	N/A	x	N/A	x	x	N/A	N/A	N/A	N/A
A10BD25	Metforminum + Saxagliptinum + Dapagliflozinum	N/A	N/A	N/A	N/A	N/A	N/A	x	N/A	N/A	N/A	N/A	N/A	N/A	N/A
A10BF01	Acarbosum	x	x	x	x	x	x	x	x	x	x	x	N/A	x	x

A10BG03	Pioglitazonum	x	x	x	x	x	N/A	x	x	x	x	x	x	x	x
A10BH01	Sitagliptinum	x	x	x	x	x	x	x	x	x	x	x	x	x	x
A10BH02	Vildagliptinum	x	x	x	x	x	x	x	x	x	x	x	x	x	x
A10BH03	Saxagliptinum	N/A	N/A	x	x	x	x	x	N/A	x	x	x	x	x	x
A10BH04	Alogliptinum	N/A	N/A	x	x	x	N/A	x	N/A	N/A	N/A	x	x	x	x
A10BH05	Linagliptinum	x	x	x	x	x	N/A	x	x	x	N/A	x	x	x	x
A10BJ01	Exenatidum	x	x	x	x	x	x	x	N/A	x	x	x	x	x	x
A10BJ02	Liraglutidum	x	x	x	x	x	x	x	N/A	x	x	x	x	x	x
A10BJ03	Lixisenatidum	x	x	x	N/A	x	N/A	x	N/A	x	x	x	x	x	x
A10BJ05	Dulaglutidum	x	x	x	x	x	x	x	x	x	x	x	x	x	x
A10BJ06	Semaglutidum	x	x	x	x	x	x	x	x	x	x	x	x	x	x
A10BK01	Dapagliflozinum	x	x	x	x	x	x	x	x	x	x	x	x	x	x
A10BK02	Canagliflozinum	x	x	x	x	x	N/A	x	x	x	N/A	N/A	N/A	x	x
A10BK03	Empagliflozinum	x	x	x	x	x	x	x	x	x	x	x	x	x	x
A10BK04	Ertugliflozinum	N/A	N/A	x	x	x	N/A	x	x	x	N/A	N/A	N/A	x	x
A10BK06	Sotagliflozin	N/A	N/A	x	N/A	N/A	N/A	x	N/A	N/A	N/A	N/A	N/A	N/A	N/A
A10BX01	Guar Gum	N/A	N/A	N/A	N/A	N/A	N/A	N/A	N/A	N/A	N/A	N/A	N/A	x	x
A10BX02	Repaglinidum	x	x	x	x	x	x	x	N/A	x	x	x	x	x	x
A10BX03	Nateglinidum	N/A	N/A	x	N/A	N/A	N/A	x	N/A	x	N/A	N/A	N/A	x	x
**Total INNs**	**44**	**27**	**27**	**41**	**33**	**37**	**22**	**41**	**15**	**35**	**27**	**25**	**24**	**39**	**38**

INN- International Non-proprietary Name; x-present active substance; N/A- not available.

Supplementary Table S2. Number of brand names for all authorized medicines compared with medicines having a pharmacy price by ATC code, INN and country

ATC code	INN	Bulgaria		Czech Republic		France		Polonia		Romania		Serbia		Spania	
		N	n	N	n	N	n	N	n	N	n	N	n	N	n
A10BA02	Metforminum	16	12	22	16	25	17	25	14	9	8	7	7	21	18
A10BB01	Glibenclamidum	N/A	N/A	1	N/A	7	6	1	N/A	1	1	1	1	2	1
A10BB07	Glipizidum	N/A	N/A	1	1	3	1	2	1	1	1	N/A	N/A	1	1
A10BB08	Gliquidonum	N/A	N/A	1	1	N/A	N/A	1	N/A	1	1	N/A	N/A	N/A	N/A
A10BB09	Gliclazidum	11	8	4	4	20	18	14	11	4	3	8	7	16	14
A10BB12	Glimepiridum	6	5	6	6	13	12	12	8	1	1	5	5	15	15
A10BB91	Glisentidum	N/A	N/A	N/A	N/A	N/A	N/A	N/A	N/A	N/A	N/A	N/A	N/A	1	1
A10BD02	Metforminum + Glibenclamidum	N/A	N/A	1	1	1	1	N/A	N/A	3	3	N/A	N/A	N/A	N/A
A10BD05	Pioglitazonum + Metforminum	1	1	4	1	1	N/A	2	N/A	2	N/A	N/A	N/A	3	3
A10BD06	Pioglitazonum + Glimepiridum	N/A	N/A	1	N/A	1	N/A	1	N/A	N/A	N/A	N/A	N/A	1	1
A10BD07	Sitagliptinum + Metforminum	3	1	9	3	20	2	15	N/A	5	1	5	4	26	4
A10BD08	Vildagliptinum + Metforminum	5	3	11	1	14	8	13	N/A	7	4	1	1	17	3
A10BD09	Pioglitazonum + Alogliptinum	N/A	N/A	1	1	N/A	N/A	1	N/A	N/A	N/A	N/A	N/A	1	1
A10BD10	Saxagliptinum + Metforminum	1	1	1	1	1	1	1	N/A	1	1	1	1	1	1
A10BD11	Linagliptinum + Metforminum	1	1	1	1	N/A	N/A	1	N/A	1	N/A	N/A	N/A	1	1
A10BD13	Metforminum + Alogliptinum	N/A	N/A	1	1	1	N/A	1	N/A	N/A	N/A	N/A	N/A	1	1
A10BD15	Dapagliflozinum+ Metforminum	1	1	2	1	1	1	2	N/A	1	1	1	1	2	2
A10BD16	Canagliflozinum + Metforminum	1	1	1	1	1	N/A	1	N/A	1	N/A	N/A	N/A	1	1
A10BD19	Linagliptinum+Empagliflozinum	1	1	1	N/A	1	N/A	1	N/A	N/A	N/A	N/A	N/A	1	1
A10BD20	Empagliflozinum +Metforminum	1	1	1	1	1	1	1	N/A	1	1	1	1	1	1
A10BD21	Saxagliptinum +Dapagliflozinum	1	1	1	N/A	1	N/A	1	N/A	1	1	1	1	1	N/A
A10BD23	Ertugliflozinum + Metforminum	N/A	N/A	1	1	1	N/A	1	N/A	1	N/A	N/A	N/A	1	1
A10BD24	Ertugliflozinum + Sitagliptinum	N/A	N/A	1	N/A	1	N/A	1	N/A	1	1	N/A	N/A	N/A	N/A

A10BD25	Metforminum+Saxagliptinum+ Dapagliflozinum	N/A	N/A	N/A	N/A	N/A	N/A	1	N/A	N/A	N/A	N/A	N/A	N/A	N/A
A10BF01	Acarbosum	2	2	2	1	8	7	4	2	1	1	1	N/A	6	6
A10BG03	Pioglitazonum	3	2	6	6	1	N/A	8	1	3	1	1	1	6	5
A10BH01	Sitagliptinum	5	1	15	2	21	12	25	1	9	1	3	3	28	4
A10BH02	Vildagliptinum	7	2	16	1	15	8	13	1	9	4	4	3	22	3
A10BH03	Saxagliptinum	N/A	N/A	1	1	2	1	1	N/A	1	1	1	1	1	1
A10BH04	Alogliptinum	N/A	N/A	1	1	1	N/A	1	N/A	N/A	N/A	1	1	1	1
A10BH05	Linagliptinum	1	1	3	1	2	N/A	4	1	1	N/A	1	1	3	1
A10BJ01	Exenatidum	1	1	2	2	2	1	2	N/A	2	2	1	1	2	2
A10BJ02	Liraglutidum	1	1	2	1	2	2	2	N/A	2	2	1	1	2	1
A10BJ03	Lixisenatidum	1	1	1	N/A	1	N/A	1	N/A	1	1	1	1	1	1
A10BJ05	Dulaglutidum	1	1	1	1	1	1	1	1	1	1	1	1	1	1
A10BJ06	Semaglutidum	1	1	3	2	2	1	3	1	2	2	2	2	2	2
A10BK01	Dapagliflozinum	1	1	2	1	1	1	2	1	1	1	1	1	2	2
A10BK02	Canagliflozinum	1	1	1	1	1	N/A	1	1	1	N/A	N/A	N/A	1	1
A10BK03	Empagliflozinum	1	1	1	1	1	1	1	1	1	1	1	1	1	1
A10BK04	Ertugliflozinum	N/A	N/A	1	1	1	N/A	1	1	1	N/A	N/A	N/A	1	1
A10BK06	Sotagliflozin	N/A	N/A	1	N/A	N/A	N/A	1	N/A	N/A	N/A	N/A	N/A	N/A	N/A
A10BX01	Guar Gum	N/A	N/A	N/A	N/A	N/A	N/A	N/A	N/A	N/A	N/A	N/A	N/A	1	1
A10BX02	Repaglinidum	3	3	8	5	15	13	6	N/A	5	3	3	2	17	17
A10BX03	Nateglinidum	N/A	N/A	1	N/A	N/A	N/A	1	N/A	1	N/A	N/A	N/A	1	1
**Total number of brand names**		**78**	**56**	**141**	**70**	**192**	**116**	**177**	**46**	**84**	**49**	**54**	**49**	**213**	**123**

INN- International Non-proprietary Name; N- authorized antidiabetic medicines; n- antidiabetic medicines having a pharmacy price; N/A- not available.

Supplementary Table S3. Overview of the common brand names (all approved medicines) in the countries of study

ATC code	INN	Total number of common brand names (≥ 2)
A10BA02	Metforminum	15
Details	Bulgaria, Czech Republic, Poland, Romania, Serbia - 3 (i + 2 g); Bulgaria, Czech Republic, Romania, Spain - 1 (g); Bulgaria, Serbia- 1 (g); Bulgaria, Czech Republic, Spain - 1 (g); Bulgaria, Czech Republic, Poland - 1 (g) Czech Republic, France- 1 (g); Czech Republic, Poland- 1 (g); Czech Republic, Spain- 1 (g) Czech Republic, France, Spain-2 (g); France, Romania- 1 (g); France, Poland, Spain -1 (g)	
A10BB01	Glibenclamidum	1
Details	Czech Republic, Poland, Spain - 1 (i)	
A10BB08	Gliquidonum	1
Details	Czech Republic, Poland, Romania - 1 (i)	
A10BB09	Gliclazidum	10
Details	Bulgaria, Czech Republic, Poland, Romania, Serbia -2 (i+g); Bulgaria, France, Poland - 1 (g) France, Poland, Spain- 2 (i+g); France, Spain- 5 (g)	
A10BB12	Glimepiridum	5
Details	Bulgaria, Czech Republic, Poland, Romania, Serbia, Spain -1 (i); Bulgaria, Serbia- 1 (g) Czech Republic, Spain- 1 (g); Czech Republic, Spain- 1 (g); Czech Republic, Poland, Spain- 1 (g)	
A10BD02	Metforminum + Glibenclamidum	2
Details	Czech Republic, Romania- 1 (i); France Romania- 1 (i)	
A10BD05	Pioglitazonum+ Metforminum	2
Details	Czech Republic,France, Poland, Romania, Spain- 1 (i); Czech Republic, Poland, Romania, Spain- 1 (i)	
A10BD06	Pioglitazonum+ Glimepiridum	1
Details	Czech Republic, Poland, Spain, France- 1 (i)	
A10BD07	Sitagliptinum + Metforminum	16
Details	Bulgaria, Czech Republic, France, Poland, Romania, Serbia, Spain- 1 (i); Bulgaria, Czech Republic, Poland, Romania, Serbia- 1 (g) Czech Republic, Poland, Romania, Spain- 1 (i); Bulgaria, Czech Republic, Poland- 1 (g); Czech Republic, Poland, Spain- 1 (i)	

	Czech Republic, France, Poland- 1 (g); Czech Republic, Poland- 1 (g); Czech Republic, France, Poland, Spain- 1 (i) Poland, Romania, Spain- 1 (g); Poland, Spain- 2 (g); Poland, Romania- 1 (g); France, Poland- 2 (g); France, Poland- 2 (g); France, Spain- 2 (g)	
A10BD08	Vildagliptinum + Metforminum	17
Details	Bulgaria, Czech Republic, France, Poland, Romania, Spain- 1 (i); Bulgaria, Czech Republic, Romania, Spain- 1 (g) Bulgaria, Czech Republic, Poland, Romania- 1 (g) Czech Republic, France, Poland, Romania, Spain- 2 (i); Czech Republic, Poland, Spain- 2 (g); Czech Republic, France, Spain- 2 (g) Bulgaria, Poland- 1 (g); Czech Republic, Poland- 1 (g); Poland, Romania- 2 (g); Bulgaria, Poland, Spain- 1 (g) Czech Republic, Spain- 1 (g); France, Spain - 2 (g)	
A10BD09	Pioglitazonum + Alogliptinum	1
Details	Czech Republic, Poland, Spain- 1 (i)	
A10BD10	Saxagliptinum + Metforminum	1
Details	Bulgaria, Czech Republic, France, Poland, Romania, Serbia, Spain- 1 (i)	
A10BD11	Linagliptinum+Metforminum	1
Details	Bulgaria, Czech Republic, France, Poland, Romania, Spain- 1 (i)	
A10BD13	Metforminum + Alogliptinum	1
Details	Czech Republic, France, Poland, Spain- 1 (i)	
A10BD15	Dapagliflozinum+Metforminum	2
Details	Czech Republic, Poland, Spain- 1 (i); Bulgaria, Czech Republic, France, Poland, Romania, Serbia, Spain- 1 (i)	
A10BD16	Canagliflozinum + Metforminum	1
Details	Bulgaria, Czech Republic, France, Poland, Romania, Spain- 1 (i)	
A10BD19	Linagliptinum + Empagliflozinum	1
Details	Bulgaria, Czech Republic, France, Poland, Spain- 1 (i)	
A10BD20	Empagliflozinum+Metforminum	1
Details	Bulgaria, Czech Republic, France, Poland, Romania, Serbia, Spain- 1 (i)	
A10BD21	Saxagliptinum+ Dapagliflozinum	1
Details	Bulgaria, Czech Republic, France, Poland, Romania, Serbia, Spain- 1 (i)	
A10BD23	Ertugliflozinum + Metforminum	1
Details	Czech Republic, France, Poland, Romania, Spain- 1 (i)	

A10BD24	Ertugliflozinum + Sitagliptinum	1
Details	Czech Republic, France, Poland, Romania- 1 (i)	
A10BF01	Acarbosum	3
Details	Bulgaria, Czech Republic, Poland, Romania, Serbia, Spain- 1 (i); Czech Republic, Spain- 1 (g); Czech Republic, Poland- 1 (g)	
A10BG03	Pioglitazonum	5
Details	Bulgaria, Czech Republic, Poland, Romania, Spain- 1 (g); Czech Republic, France, Poland, Romania, Spain- 1 (i); Czech Republic, Poland, Spain- 2 (g); Poland, Romania- 1 (g)	
A10BH01	Sitagliptinum	22
Details	Bulgaria, Czech Republic, France, Poland, Romania, Serbia, Spain - 1 (i); Bulgaria, Czech Republic, Poland, Romania, Serbia- 1 (g); Bulgaria, Czech Republic, France, Poland, Romania, Spain- 1 (g); Bulgaria, Czech Republic, Poland, Romania- 1 (g); Czech Republic, France, Poland, Spain- 2 (i+g); Czech Republic, Poland, Spain- 3 (2i+g); Poland, Romania, Spain- 1 (g); Czech Republic, Poland- 3 (g); France, Spain - 2 (g); Poland, Spain- 3 (g); Poland, Romania - 1 (g); Romania, Spain - 1 (g); Bulgaria, Czech Republic - 1 (g); France, Poland – 1 (g)	
A10BH02	Vildagliptinum	20
Details	Bulgaria, Czech Republic, France, Poland, Romania, Serbia, Spain- 1 (i); Bulgaria, Czech Republic, Romania, Serbia, Spain- 1 (g); Bulgaria, Czech Republic, Romania- 1 (g); Czech Republic, France, Poland, Romania, Spain- 2 (i); Bulgaria, Czech Republic, Poland- 1 (g); Czech Republic, Poland, Serbia- 1 (g); Czech Republic, Poland, Romania- 1 (g); Czech Republic, France, Spain - 4 (g); Bulgaria, Poland- 1 (g); Czech Republic, Poland- 2 (g); Czech Republic, Spain- 1 (g); France, Spain- 2 (g); Poland, Romania- 2 (g)	
A10BH03	Saxagliptinum	1
Details	Czech Republic, France, Poland, Romania, Serbia, Spain- 1 (i)	
A10BH04	Alogliptinum	1
Details	Czech Republic, France, Poland, Serbia, Spain- 1 (i)	
A10BH05	Linagliptinum	4
Details	Bulgaria, Czech Republic, France, Poland, Romania, Serbia, Spain- 1 (i); Czech Republic, Spain- 1 (g) Czech Republic, Poland- 1 (g); Poland, Spain- 1 (g)	
A10BJ01	Exenatidum	2
Details	Czech Republic, France, Poland, Romania, Spain- 1 (i); Bulgaria, Czech Republic, France, Poland, Romania, Serbia, Spain- 1 (i)	
A10BJ02	Liraglutidum	2
Details	Czech Republic, France, Poland, Romania, Spain- 1 (i); Bulgaria, Czech Republic, France, Poland, Romania, Serbia, Spain- 1 (i)	

A10BJ03	Lixisenatidum	1
Details	Bulgaria, Czech Republic, France, Poland, Romania, Serbia, Spain- 1 (i)	
A10BJ05	Dulaglutidum	1
Details	Bulgaria, Czech Republic, France, Poland, Romania, Serbia, Spain- 1 (i)	
A10BJ06	Semaglutidum	3
Details	Bulgaria, Czech Republic, France, Poland, Romania, Serbia, Spain- 1 (i); Czech Republic, France, Poland, Romania, Serbia, Spain- 1 (i); Czech Republic, Poland- 1 (i)	
A10BK01	Dapagliflozinum	2
Details	Bulgaria, Czech Republic, France, Poland, Romania, Serbia, Spain- 1 (i); Czech Republic, Poland, Spain- 1 (i)	
A10BK02	Canagliflozinum	1
Details	Bulgaria, Czech Republic, France, Poland, Romania, Spain- 1 (i)	
A10BK03	Empagliflozinum	1
Details	Bulgaria, Czech Republic, France, Poland, Romania, Serbia, Spain- 1 (i)	
A10BK04	Ertugliflozinum	1
Details	Czech Republic, France, Poland, Romania, Spain- 1 (i)	
A10BK06	Sotagliflozin	1
Details	Czech Republic Poland- 1 (i)	
A10BX02	Repaglinidum	7
Details	Bulgaria, Czech Republic, France, Poland, Romania, Spain- 1 (g); Czech Republic, France, Poland, Serbia, Spain- 1 (g) Czech Republic, France, Poland, Romania, Spain- 1 (g); Czech Republic, France, Poland, Spain- 2 (i+g); Czech Republic, Poland, Spain- 1 (i); France, Spain- 1 (g)	
A10BX03	Nateglinidum	1
Details	Czech Republic, Poland, Romania, Spain- 1 (i)	

INN- International Non-proprietary Name; i- innovative medicine; g-generic medicine.

Supplementary Table S4. Overview of the common brand names (having a pharmacy price) in the countries of study

ATC code	INN	Total number of common brand names (≥ 2)
A10BA02	Metforminum	11
Details	Bulgaria, Czech Republic, France, Poland, Romania, Serbia - 1 (i); Bulgaria, Czech Republic, Poland, Romania, Serbia - 2 (i+ g) Bulgaria, Czech Republic, Romania, Serbia - 1 (g); Bulgaria, Serbia- 1 (g); Czech Republic, France- 1 (g); Czech Republic, Poland- 1 (g); Czech Republic, Spain- 1 (g); Czech Republic, France, Spain-2 (g); France, Poland, Spain -1 (g)	
A10BB08	Gliquidonum	1
Details	Czech Republic, Romania - 1 (i)	
A10BB09	Gliclazidum	7
Details	Bulgaria, Czech Republic, Poland, Romania, Serbia -1 (i); Czech Republic, Poland, Romania, Serbia - 1 (g) France, Spain- 5 (g)	
A10BB12	Glimepiridum	5
Details	Bulgaria, Czech Republic, Poland, Romania, Serbia, Spain -1 (i); Bulgaria, Serbia- 1 (g) Czech Republic, Spain- 2 (g); Czech Republic, France, Spain- 1 (g);	
A10BD02	Metforminum + Glibenclamidum	2
Details	Czech Republic, Romania- 1 (i); France Romania- 1 (i)	
A10BD05	Pioglitazonum + Metforminum	1
Details	Czech Republic, Spain- 1 (i)	
A10BD06	Pioglitazonum + Glimepiridum	N/A
A10BD07	Sitagliptinum + Metforminum	3
Details	Bulgaria, Czech Republic, France, Romania, Serbia, Spain - 1 (i); Czech Republic, France, Spain- 1 (i) Czech Republic, Spain- 1 (i)	
A10BD08	Vildagliptinum + Metforminum	2
Details	Bulgaria, Czech Republic, France, Romania, Spain- 1 (i); Bulgaria, Romania- 1 (g)	
A10BD09	Pioglitazonum + Alogliptinum	1
Details	Czech Republic, Spain- 1 (i)	
A10BD10	Saxagliptinum + Metforminum	1
Details	Bulgaria, Czech Republic, France, Romania, Serbia, Spain- 1 (i)	

A10BD11	Linagliptinum + Metforminum	1
Details	Bulgaria, Czech Republic, Spain- 1 (i)	
A10BD13	Metforminum + Alogliptinum	1
Details	Czech Republic, Spain- 1 (i)	
A10BD15	Dapagliflozinum+Metforminum	1
Details	Bulgaria, Czech Republic, France, Romania, Serbia, Spain- 1 (i)	
A10BD16	Canagliflozinum+Metforminum	1
Details	Bulgaria, Czech Republic, Spain- 1 (i)	
A10BD19	Linagliptinum+Empagliflozinum	1
Details	Bulgaria, Spain- 1 (i)	
A10BD20	Empagliflozinum+Metforminum	1
Details	Bulgaria, Czech Republic, France, Romania, Serbia, Spain- 1 (i)	
A10BD21	Saxagliptinum+Dapagliflozinum	1
Details	Bulgaria, Poland, Romania, Serbia- 1 (i)	
A10BD23	Ertugliflozinum + Metforminum	1
Details	Czech Republic, Spain- 1 (i)	
A10BD24	Ertugliflozinum + Sitagliptinum	N/A
A10BF01	Acarbosum	1
Details	Bulgaria, Poland, Romania, Spain- 1 (i); France, Spain- 1 (g)	
A10BG03	Pioglitazonum	2
Details	Bulgaria, Czech Republic, Romania, Spain- 1 (g); Czech Republic, Spain- 1 (i)	
A10BH01	Sitagliptinum	3
Details	Bulgaria, Czech Republic, France, Romania, Serbia, Spain - 1 (i); Czech Republic, Spain- 1 (i); France, Spain - 1 (i); Poland, Spain- 1(i)	
A10BH02	Vildagliptinum	3
Details	Bulgaria, Czech Republic, France, Romania, Serbia, Spain- 1 (i); Bulgaria, Romania- 1 (g)<br>Czech Republic, Poland, Romania, Spain- 2 (i)	
A10BH03	Saxagliptinum	1
Details	Czech Republic, France, Poland, Romania, Serbia, Spain- 1 (i)	
A10BH04	Alogliptinum	1

Details	Czech Republic, Poland, Serbia, Spain- 1 (i)	
A10BH05	Linagliptinum	4
Details	Bulgaria, Czech Republic, Poland, Serbia, Spain- 1 (i)	
A10BJ01	Exenatidum	2
Details	Czech Republic, Romania, Spain- 1 (i); Bulgaria, Czech Republic, France, Romania, Serbia, Spain- 1 (i)	
A10BJ02	Liraglutidum	2
Details	Bulgaria, Czech Republic, France, Romania, Serbia, Spain- 1 (i); France, Romania- 1 (i)	
A10BJ03	Lixisenatidum	1
Details	Bulgaria, Czech Republic, Poland, Romania, Serbia, Spain- 1 (i)	
A10BJ05	Dulaglutidum	1
Details	Bulgaria, Czech Republic, France, Poland, Romania, Serbia, Spain- 1 (i)	
A10BJ06	Semaglutidum	2
Details	Bulgaria, Czech Republic, France, Poland, Romania, Serbia, Spain- 1 (i); Czech Republic, Romania, Serbia, Spain- 1 (i)	
A10BK01	Dapagliflozinum	1
Details	Bulgaria, Czech Republic, France, Poland, Romania, Serbia, Spain- 1 (i)	
A10BK02	Canagliflozinum	1
Details	Bulgaria, Czech Republic, Poland, Spain- 1 (i)	
A10BK03	Empagliflozinum	1
Details	Bulgaria, Czech Republic, France, Poland, Romania, Serbia, Spain- 1 (i)	
A10BK04	Ertugliflozinum	1
Details	Czech Republic, Poland, Spain- 1 (i)	
A10BK06	Sotagliflozin	N/A
A10BX02	Repaglinidum	6
Details	Bulgaria, Czech Republic, France, Romania, Spain- 1 (g); Czech Republic, Spain- 1 (g)<br>Czech Republic, France, Spain- 1 (i); France, Spain- 3 (g)	
A10BX03	Nateglinidum	N/A

INN- International Non-proprietary Name; i=innovative medicine; g=generic medicine; N/A-no common brand name available

Supplementary Table S5. Number of innovator and generic brand names per INN for all authorized medicines

ATC code	INN	Number of innovator brand name/INN							Number of generic brand name/INN						
		**B**	**CR**	**F**	**P**	**R**	**SB**	**SP**	**B**	**CR**	**F**	**P**	**R**	**SB**	**SP**
A10BA02	Metforminum	2	2	1	2	2	2	1	14	20	24	23	7	5	20
A10BB01	Glibenclamidum	N/A	1	2	1	0	1	2	N/A	0	5	0	1	0	0
A10BB07	Glipizidum	N/A	1	2	1	1	N/A	1	N/A	0	1	1	0	N/A	0
A10BB08	Gliquidonum	N/A	1	N/A	1	1	N/A	N/A	N/A	0	N/A	0	0	N/A	N/A
A10BB09	Gliclazidum	1	1	1	2	1	1	1	10	3	19	12	3	7	15
A10BB12	Glimepiridum	1	1	1	1	1	1	1	5	5	12	11	0	4	14
A10BB91	Glisentidum	N/A	N/A	N/A	N/A	N/A	N/A	1	N/A	N/A	N/A	N/A	N/A	**N/A**	0
A10BD02	Metforminum+ Glibenclamidum	N/A	1	1	N/A	2	N/A	N/A	N/A	0	0	N/A	1	N/A	N/A
A10BD05	Pioglitazonum + Metforminum	0	2	1	2	1	N/A	2	1	2	0	0	1	N/A	1
A10BD06	Pioglitazonum + Glimepiridum	N/A	1	1	1	N/A	N/A	1	N/A	0	0	0	N/A	N/A	0
A10BD07	Sitagliptinum+ Metforminum	1	4	2	4	2	1	4	2	5	18	11	3	4	22
A10BD08	Vildagliptinum + Metforminum	1	3	3	3	3	0	3	4	8	11	10	4	1	14
A10BD09	Pioglitazonum + Alogliptinum	N/A	1	N/A	1	N/A	N/A	1	N/A	0	N/A	0	N/A	N/A	0
A10BD10	Saxagliptinum + Metforminum	1	1	1	1	1	1	1	0	0	0	0	0	0	0
A10BD11	Linagliptinum+ Metforminum	1	1	1	1	1	N/A	1	0	0	0	0	0	N/A	0
A10BD13	Metforminum + Alogliptinum	N/A	1	1	1	N/A	N/A	1	N/A	0	0	0	N/A	N/A	0
A10BD15	Dapagliflozinum+Metforminum	1	2	1	2	1	1	2	0	0	0	0	0	0	0
A10BD16	Canagliflozinum + Metforminum	1	1	1	1	1	N/A	1	0	0	0	0	0	N/A	0
A10BD19	Linagliptinum + Empagliflozinum	1	1	1	1	N/A	N/A	1	0	0	0	0	N/A	N/A	0
A10BD20	Empagliflozinum+Metforminum	1	1	1	1	1	1	1	0	0	0	0	0	0	0
A10BD21	Saxagliptinum+ Dapagliflozinum	1	1	1	1	1	1	1	0	0	0	0	0	0	0
A10BD23	Ertugliflozinum + Metforminum	N/A	1	1	1	1	N/A	1	N/A	0	0	0	0	N/A	0
A10BD24	Ertugliflozinum + Sitagliptinum	N/A	1	1	1	1	N/A	N/A	N/A	0	0	0	0	N/A	N/A
A10BD25	Metforminum + Saxagliptinum + Dapagliflozinum	N/A	N/A	N/A	1	N/A	N/A	N/A	N/A	N/A	N/A	0	N/A	N/A	N/A
A10BF01	Acarbosum	1	0	1	1	1	1	1	1	2	7	3	0	0	5

A10BG03	Pioglitazonum	0	1	1	2	1	0	1	3	5	0	6	2	1	5
A10BH01	Sitagliptinum	1	4	2	4	1	1	4	4	11	19	21	8	2	24
A10BH02	Vildagliptinum	1	3	3	3	3	1	3	6	13	12	10	6	3	19
A10BH03	Saxagliptinum	N/A	1	1	1	1	1	1	N/A	0	1	0	0	0	0
A10BH04	Alogliptinum	N/A	1	1	1	N/A	1	1	N/A	0	0	0	N/A	0	0
A10BH05	Linagliptinum	1	1	1	1	1	1	1	0	2	1	3	0	0	2
A10BJ01	Exenatidum	1	2	2	2	2	1	2	0	0	0	0	0	0	0
A10BJ02	Liraglutidum	1	2	2	2	2	1	2	0	0	0	0	0	0	0
A10BJ03	Lixisenatidum	1	1	1	1	1	1	1	0	0	0	0	0	0	0
A10BJ05	Dulaglutidum	1	1	1	1	1	1	1	0	0	0	0	0	0	0
A10BJ06	Semaglutidum	1	3	2	3	2	2	2	0	0	0	0	0	0	0
A10BK01	Dapagliflozinum	1	2	1	2	1	1	2	0	0	0	0	0	0	0
A10BK02	Canagliflozinum	1	1	1	1	1	N/A	1	0	0	0	0	0	N/A	0
A10BK03	Empagliflozinum	1	1	1	1	1	1	1	0	0	0	0	0	0	0
A10BK04	Ertugliflozinum	N/A	1	1	1	1	N/A	1	N/A	0	0	0	0	N/A	0
A10BK06	Sotagliflozin	N/A	1	N/A	1	N/A	N/A	N/A	N/A	0	N/A	0	N/A	N/A	N/A
A10BX01	Guar Gum	N/A	N/A	N/A	N/A	N/A	N/A	1	N/A	N/A	N/A	N/A	N/A	N/A	0
A10BX02	Repaglinidum	0	2	1	2	0	0	2	3	6	14	4	5	3	15
A10BX03	Nateglinidum	N/A	1	N/A	1	1	N/A	1	N/A	0	N/A	0	0	N/A	0

INN- International Non-proprietary Name; Countries: B-Bulgaria, CR-Czech Republic, F-France, P-Poland, R-Romania, SB-Serbia, SP-Spain
N/A-not available, there is no authorized medicine

Supplementary Table S6. Number of innovator and generic brand names per INN for medicines with pharmacy price

ATC code	INN	Number of innovator brand name/INN							Number of generic brand name/INN						
		B	CR	F	P	R	SB	SP	B	CR	F	P	R	SB	SP
A10BA02	Metforminum	2	2	1	2	2	2	1	10	14	16	12	6	5	17
A10BB01	Glibenclamidum	N/A	N/A	1	N/A	0	1	1	N/A	N/A	5	N/A	1	0	0
A10BB07	Glipizidum	N/A	1	1	0	1	N/A	1	N/A	0	0	1	0	N/A	0
A10BB08	Gliquidonum	N/A	1	N/A	N/A	1	N/A	N/A	N/A	0	N/A	N/A	0	N/A	N/A
A10BB09	Gliclazidum	1	1	1	1	1	1	1	7	3	17	10	2	6	13
A10BB12	Glimepiridum	1	1	1	1	1	1	1	4	5	11	7	0	4	14
A10BB91	Glisentidum	N/A	N/A	N/A	N/A	N/A	N/A	1	N/A	N/A	N/A	N/A	N/A	N/A	0
A10BD02	Metforminum+ Glibenclamidum	N/A	1	1	N/A	2	N/A	N/A	N/A	0	0	N/A	1	N/A	N/A
A10BD05	Pioglitazonum + Metforminum	0	1	N/A	N/A	N/A	N/A	2	1	0	N/A	N/A	N/A	N/A	1
A10BD06	Pioglitazonum + Glimepiridum	N/A	N/A	N/A	N/A	N/A	N/A	1	N/A	N/A	N/A	N/A	N/A	N/A	0
A10BD07	Sitagliptinum+ Metforminum	1	3	2	N/A	1	1	4	0	0	0	N/A	0	3	0
A10BD08	Vildagliptinum + Metforminum	1	1	1	N/A	1	0	3	2	0	7	N/A	3	1	0
A10BD09	Pioglitazonum + Alogliptinum	N/A	1	N/A	N/A	N/A	N/A	1	N/A	0	N/A	N/A	N/A	N/A	0
A10BD10	Saxagliptinum + Metforminum	1	1	1	N/A	1	1	1	0	0	0	N/A	0	0	0
A10BD11	Linagliptinum+ Metforminum	1	1	N/A	N/A	N/A	N/A	1	0	0	N/A	N/A	N/A	N/A	0
A10BD13	Metforminum + Alogliptinum	N/A	1	N/A	N/A	N/A	N/A	1	N/A	0	N/A	N/A	N/A	N/A	0
A10BD15	Dapagliflozinum+Metforminum	1	1	1	N/A	1	1	2	0	0	0	N/A	0	0	0
A10BD16	Canagliflozinum + Metforminum	1	1	N/A	N/A	N/A	N/A	1	0	0	N/A	N/A	N/A	N/A	0
A10BD19	Linagliptinum+ Empagliflozinum	1	N/A	N/A	N/A	N/A	N/A	1	0	N/A	N/A	N/A	N/A	N/A	0
A10BD20	Empagliflozinum+Metforminum	1	1	1	N/A	1	1	1	0	0	0	N/A	0	0	0
A10BD21	Saxagliptinum+ Dapagliflozinum	1	N/A	N/A	N/A	1	1	N/A	0	N/A	N/A	N/A	0	0	N/A
A10BD23	Ertugliflozinum + Metforminum	N/A	1	N/A	N/A	N/A	N/A	1	N/A	0	N/A	N/A	N/A	N/A	0
A10BD24	Ertugliflozinum + Sitagliptinum	N/A	N/A	N/A	N/A	1	N/A	N/A	N/A	N/A	N/A	N/A	0	N/A	N/A
A10BD25	Metforminum + Saxagliptinum + Dapagliflozinum	N/A	N/A	N/A	N/A	N/A	N/A	N/A	N/A	N/A	N/A	N/A	N/A	N/A	N/A
A10BF01	Acarbosum	1	0	1	1	1	N/A	1	1	1	6	1	0	N/A	5
A10BG03	Pioglitazonum	0	1	N/A	0	0	0	1	2	5	N/A	1	1	1	4

A10BH01	Sitagliptinum	1	2	2	1	1	1	4	0	0	10	0	0	2	0
A10BH02	Vildagliptinum	1	1	1	0	1	1	3	1	0	7	1	3	2	0
A10BH03	Saxagliptinum	N/A	1	1	N/A	1	0	1	N/A	0	0	N/A	0	1	0
A10BH04	Alogliptinum	N/A	1	N/A	N/A	N/A	1	1	N/A	0	N/A	N/A	N/A	0	0
A10BH05	Linagliptinum	1	1	N/A	1	N/A	1	1	0	0	N/A	0	N/A	0	0
A10BJ01	Exenatidum	1	2	1	N/A	2	1	2	0	0	0	N/A	0	0	0
A10BJ02	Liraglutidum	1	1	2	N/A	2	1	1	0	0	0	N/A	0	0	0
A10BJ03	Lixisenatidum	1	N/A	N/A	N/A	1	1	1	0	N/A	N/A	N/A	0	0	0
A10BJ05	Dulaglutidum	1	1	1	1	1	1	1	0	0	0	0	0	0	0
A10BJ06	Semaglutidum	1	2	1	1	2	2	2	0	0	0	0	0	0	0
A10BK01	Dapagliflozinum	1	1	1	1	1	1	2	0	0	0	0	0	1	0
A10BK02	Canagliflozinum	1	1	N/A	1	N/A	N/A	1	0	0	N/A	0	N/A	N/A	0
A10BK03	Empagliflozinum	1	1	1	1	1	1	1	0	0	0	0	0	0	0
A10BK04	Ertugliflozinum	N/A	1	N/A	1	N/A	N/A	1	N/A	0	N/A	0	N/A	N/A	0
A10BK06	Sotagliflozin	N/A	N/A	N/A	N/A	N/A	N/A	N/A	N/A	N/A	N/A	N/A	N/A	N/A	N/A
A10BX01	Guar Gum	N/A	N/A	N/A	N/A	N/A	N/A	1	N/A	N/A	N/A	N/A	N/A	N/A	0
A10BX02	Repaglinidum	0	1	1	N/A	0	0	2	3	4	12	N/A	3	2	15
A10BX03	Nateglinidum	N/A	N/A	N/A	N/A	N/A	N/A	1	N/A	N/A	N/A	N/A	N/A	N/A	0

INN- International Non-proprietary Name; Countries: B-Bulgaria, CR-Czech Republic, F-France, P-Poland, R-Romania, SB-Serbia, SP-Spain
N/A-not available, there is no medicine having a pharmacy price (innovator and generic medicine)

Supplementary Table S7. Types of pharmaceutical forms

ATC code	INN	All authorized medicines							Medicines having a pharmacy price						
		**B**	**CR**	**F**	**P**	**R**	**SB**	**SP**	**B**	**CR**	**F**	**P**	**R**	**SB**	**SP**
A10BA02	Metforminum	1, 4	1, 4	1, 2, 4, 10	1, 4, 2	1, 4, 2	1, 4	1	1, 4	1, 4	1, 2	1, 4	1, 4	1, 4	1
A10BB01	Glibenclamidum	N/A	7	2, 7	7	2	2	2, 7	N/A	N/A	2	N/A	2	2	2
A10BB07	Glipizidum	N/A	2	2, 4	2, 4	3	N/A	2	N/A	2	4	2	3	N/A	2
A10BB08	Gliquidonum	N/A	2	N/A	2	2	N/A	N/A	N/A	2	N/A	N/A	2	N/A	N/A
A10BB09	Gliclazidum	2, 3, 4	3	2, 3	2, 3, 4	2, 3, 4	2, 3, 4	3	2, 3, 4	3	2, 3	2, 3, 4	2, 3, 4	2, 3, 4	3
A10BB12	Glimepiridum	2	2	2	2	2	2	2	2	2	2	2	2	2	2
A10BB91	Glisentidum	N/A	N/A	N/A	N/A	N/A	N/A	1	N/A	N/A	N/A	N/A	N/A	N/A	1
A10BD02	Metforminum+ Glibenclamidum	N/A	1	1	N/A	1, 9	N/A	N/A	N/A	1	1	N/A	1, 9	N/A	N/A
A10BD05	Pioglitazonum + Metforminum	1	1	1	1	1	N/A	1	1	1	N/A	N/A	N/A	N/A	1
A10BD06	Pioglitazonum + Glimepiridum	N/A	2	2	2	N/A	N/A	2	N/A	N/A	N/A	N/A	N/A	N/A	2
A10BD07	Sitagliptinum+ Metforminum	1	1	1	1	1	1	1, 3	1	1	1	N/A	1	1	1
A10BD08	Vildagliptinum + Metforminum	1	1	1	1	1	1	1	1	1	1	N/A	1	1	1
A10BD09	Pioglitazonum + Alogliptinum	N/A	1	N/A	1	N/A	N/A	1	N/A	1	N/A	N/A	N/A	N/A	1
A10BD10	Saxagliptinum + Metforminum	1	1	1	1	1	1	1	1	1	1	N/A	1	N/A	1
A10BD11	Linagliptinum+ Metforminum	1	1	1	1	1	N/A	1	1	1	N/A	N/A	N/A	N/A	1
A10BD13	Metforminum + Alogliptinum	N/A	1	1	1	N/A	N/A	1	N/A	1	N/A	N/A	N/A	N/A	1
A10BD15	Dapagliflozinum+Metforminum	1	1	1	1	1	1	1	1	1	1	N/A	1	1	1
A10BD16	Canagliflozinum + Metforminum	1	1	1	1	1	N/A	1	1	1	N/A	N/A	N/A	N/A	1
A10BD19	Linagliptinum+ Empagliflozinum	1	1	1	1	N/A	N/A	1	1	N/A	N/A	N/A	N/A	N/A	1
A10BD20	Empagliflozinum+Metforminum	1	1	1	1	1	1	1	1	1	1	N/A	1	1	1
A10BD21	Saxagliptinum+ Dapagliflozinum	1	1	1	1	1	1	1	1	N/A	N/A	N/A	1	1	N/A
A10BD23	Ertugliflozinum + Metforminum	N/A	1	1	1	1	N/A	1	N/A	1	N/A	N/A	N/A	N/A	1
A10BD24	Ertugliflozinum + Sitagliptinum	N/A	1	1	1	1	N/A	N/A	N/A	N/A	N/A	N/A	1	N/A	N/A
A10BD25	Metforminum + Saxagliptinum + Dapagliflozinum	N/A	N/A	N/A	3	N/A	N/A	N/A	N/A	N/A	N/A	N/A	N/A	N/A	N/A

A10BF01	Acarbosum	2	2	2	2	2	2	2	2	2	2	2	2	N/A	2
A10BG03	Pioglitazonum	2	2	2	2	2	2	2	2	2	N/A	2	2	2	2
A10BH01	Sitagliptinum	1	1	1	1	1	1	1	1	1	1	1	1	1	1
A10BH02	Vildagliptinum	2	2	2	2	2	2	2	2	2	2	2	2	2	2
A10BH03	Saxagliptinum	N/A	1	1	1	1	1	1	N/A	1	1	N/A	1	1	1
A10BH04	Alogliptinum	N/A	1	1	1	N/A	1	1	N/A	1	N/A	N/A	N/A	1	1
A10BH05	Linagliptinum	1	1	1	1	1	1	1	1	1	N/A	1	N/A	1	1
A10BJ01	Exenatidum	5	5, 6	5, 6	5, 6	5, 6	5	5, 6	5	5, 6	5	N/A	5, 6	5	5, 6
A10BJ02	Liraglutidum	5	5	5	5	5	5	5	5	5	5	N/A	5	5	5
A10BJ03	Lixisenatidum	5	5	5	5	5	5	5	5	N/A	N/A	N/A	5	5	5
A10BJ05	Dulaglutidum	5	5	5	5	5	5	5	5	5	5	5	5	5	5
A10BJ06	Semaglutidum	5	2, 5	2, 5	2, 5	2, 5	2, 5	2, 5	5	2, 5	5	5	2, 5	2, 5	2, 5
A10BK01	Dapagliflozinum	1	1	1	1	1	1	1	1	1	1	1	1	1	1
A10BK02	Canagliflozinum	1	1	1	1	1	N/A	1	1	1	N/A	1	N/A	N/A	1
A10BK03	Empagliflozinum	1	1	1	1	1	1	1	1	1	1	1	1	1	1
A10BK04	Ertugliflozinum	N/A	1	1	1	1	N/A	1	N/A	1	N/A	1	N/A	N/A	1
A10BK06	Sotagliflozin	N/A	1	N/A	1	N/A	N/A	N/A	N/A	N/A	N/A	N/A	N/A	N/A	N/A
A10BX01	Guar Gum	N/A	N/A	N/A	N/A	N/A	N/A	8	N/A	N/A	N/A	N/A	N/A	N/A	8
A10BX02	Repaglinidum	2	2	2	2	2	2	2	2	2	2	N/A	2	2	2
A10BX03	Nateglinidum	N/A	1	N/A	1	1	N/A	1	N/A	N/A	N/A	N/A	N/A	N/A	1
**Total**	**10**	**5**	**7**	**8**	**7**	**7**	**5**	**7**	**5**	**6**	**5**	**5**	**7**	**5**	**6**

INN- International Non-proprietary Name; Countries: B-Bulgaria, CR-Czech Republic, F-France, P-Poland, R-Romania, SB-Serbia, SP-Spain
Pharmaceutical forms: 1-filmed-coated tablet, 2-tablet, 3-modified release tablet, 4-prolonged release tablet, 5-injectable solution, 6-injectable suspension, 7-oral suspension, 8-powder, 9-capsule, 10-effervescent tablet
N/A=not available

Supplementary Table S8. Number of strengths by INN and country for all authorized and marketed medicines

ATC code	INN	All authorized medicines							Marketed medicines						
		B	CR	F	P	R	SB	SP	B	CR	F	P	R	SB	SP
A10BA02	Metforminum	4	4	4	4	4	4	3	4	4	4	4	4	4	2
A10BB01	Glibenclamidum	N/A	2	4	2	3	1	2	N/A	N/A	2	N/A	1	1	1
A10BB07	Glipizidum	N/A	1	2	2	2	N/A	1	N/A	1	2	1	1	N/A	1
A10BB08	Gliquidonum	N/A	1	N/A	1	1	N/A	N/A	N/A	1	N/A	N/A	1	N/A	N/A
A10BB09	Gliclazidum	4	2	4	4	4	4	2	3	2	3	4	3	4	2
A10BB12	Glimepiridum	5	5	4	5	5	5	2	5	5	4	5	5	5	2
A10BB91	Glisentidum	N/A	N/A	N/A	N/A	N/A	N/A	1	N/A	N/A	N/A	N/A	N/A	N/A	1
A10BD02	Metforminum + Glibenclamidum	N/A	1	3	N/A	3	N/A	N/A	N/A	1	3	N/A	3	N/A	N/A
A10BD05	Pioglitazonum + Metforminum	1	1	1	1	1	N/A	1	1	1	N/A	N/A	N/A	N/A	1
A10BD06	Pioglitazonum + Glimepiridum	N/A	3	3	3	N/A	N/A	2	N/A	N/A	N/A	N/A	N/A	N/A	2
A10BD07	Sitagliptinum+ Metforminum	2	2	2	2	2	2	4	2	2	1	N/A	1	2	1
A10BD08	Vildagliptinum + Metforminum	2	2	2	2	2	2	2	2	2	1	N/A	2	2	2
A10BD09	Pioglitazonum + Alogliptinum	N/A	3	N/A	4	N/A	N/A	2	N/A	2	N/A	N/A	N/A	N/A	2
A10BD10	Saxagliptinum + Metforminum	2	2	1	2	1	2	2	2	2	1	N/A	1	2	2
A10BD11	Linagliptinum + Metforminum	2	2	1	2	2	N/A	2	2	2	N/A	N/A	N/A	N/A	2
A10BD13	Metforminum + Alogliptinum	N/A	2	1	2	N/A	N/A	2	N/A	2	N/A	N/A	N/A	N/A	2
A10BD15	Dapagliflozinum+Metforminum	2	2	1	2	1	2	2	2	2	1	N/A	1	2	2
A10BD16	Canagliflozinum + Metforminum	2	4	4	4	4	N/A	4	2	1	N/A	N/A	N/A	N/A	4
A10BD19	Linagliptinum + Empagliflozinum	1	2	1	2	N/A	N/A	2	1	N/A	N/A	N/A	N/A	N/A	2
A10BD20	Empagliflozinum + Metforminum	2	4	2	4	4	4	4	2	2	2	N/A	2	4	2
A10BD21	Saxagliptinum + Dapagliflozinum	1	1	1	1	1	1	1	1	N/A	N/A	N/A	1	1	N/A
A10BD23	Ertugliflozinum + Metforminum	N/A	4	2	4	2	N/A	2	N/A	1	N/A	N/A	N/A	N/A	2
A10BD24	Ertugliflozinum + Sitagliptinum	N/A	2	2	2	2	N/A	N/A	N/A	N/A	N/A	N/A	2	N/A	N/A

A10BD25	Metforminum + Saxagliptinum + Dapagliflozinum	N/A	N/A	N/A	2	N/A	N/A	N/A	N/A	N/A	N/A	N/A	N/A	N/A	N/A
A10BF01	Acarbosum	1	1	2	2	2	2	2	1	1	2	2	2	N/A	2
A10BG03	Pioglitazonum	3	3	3	3	3	2	2	3	3	N/A	3	3	2	2
A10BH01	Sitagliptinum	3	3	3	3	3	3	3	1	1	2	1	2	3	3
A10BH02	Vildagliptinum	1	1	1	1	1	1	1	1	1	1	1	1	1	1
A10BH03	Saxagliptinum	N/A	2	3	2	1	2	2	N/A	1	1	N/A	1	1	2
A10BH04	Alogliptinum	N/A	3	3	3	N/A	1	3	N/A	2	N/A	N/A	N/A	1	3
A10BH05	Linagliptinum	1	1	1	1	1	1	1	1	1	N/A	1	N/A	1	1
A10BJ01	Exenatidum	1	3	3	3	3	2	3	1	3	2	N/A	3	2	3
A10BJ02	Liraglutidum	1	1	1	1	1	1	1	1	1	1	N/A	1	1	1
A10BJ03	Lixisenatidum	2	2	2	2	2	2	2	2	N/A	N/A	N/A	1	2	2
A10BJ05	Dulaglutidum	3	4	4	4	4	2	4	3	4	4	4	4	2	2
A10BJ06	Semaglutidum	3	7	6	3	6	6	6	3	6	3	3	6	6	6
A10BK01	Dapagliflozinum	1	2	1	2	2	2	2	1	2	1	2	1	2	1
A10BK02	Canagliflozinum	1	2	2	2	2	N/A	2	1	2	N/A	2	N/A	N/A	1
A10BK03	Empagliflozinum	1	2	2	2	2	2	2	1	1	2	1	2	2	2
A10BK04	Ertugliflozinum	N/A	2	1	2	2	N/A	2	N/A	1	N/A	2	N/A	N/A	2
A10BK06	Sotagliflozin	N/A	1	N/A	1	N/A	N/A	N/A	N/A	N/A	N/A	N/A	N/A	N/A	N/A
A10BX01	Guar Gum	N/A	N/A	N/A	N/A	N/A	N/A	1	N/A	N/A	N/A	N/A	N/A	N/A	1
A10BX02	Repaglinidum	4	3	3	3	4	2	3	3	3	3	N/A	3	2	3
A10BX03	Nateglinidum	N/A	3	N/A	3	3	N/A	3	N/A	N/A	N/A	N/A	N/A	N/A	3
**Total number of strengths**		**39**	**68**	**61**	**70**	**57**	**41**	**59**	**34**	**46**	**34**	**27**	**44**	**41**	**51**

INN- International Non-proprietary Name; Countries: B-Bulgaria, CR-Czech Republic, F-France, P-Poland, R-Romania, SB-Serbia, SP-Spain
N/A-not available.

Supplementary Table S9. Top 10 Marketing authorization holders for all authorized medicines

Top 10 MAHs	Bulgaria			Czech Republic			France			Poland		
	**Marketing authorization holder**	**No. of INNs**	**No. of Brand names**	**Marketing authorization holder**	**No. of INNs**	**No. of Brand names**	**Marketing authorization holder**	**No. of INNs**	**No. of Brand names**	**Marketing authorization holder**	**No. of INNs**	**No. of Brand names**
1	Tchaikapharma High Quality Medicines	5	6	AstraZeneca	6	9	Arrow Generiques	10	15	Sandoz	7	12
2	Boehringer Ingelheim	5	5	KrKa	6	7	Biogaran	10	13	AstraZeneca	7	10
3	AstraZeneca	5	5	Teva Pharmaceutical Industries Ltd	6	7	Sandoz	10	13	Accord Healthcare	7	7
4	Medochemie Ltd	5	5	Mylan	6	6	EG Labo-Laboratoires EuroGenerics	10	11	Boehringer Ingelheim	6	6
5	KrKa	4	4	Sandoz	6	6	Zentiva	10	11	Zentiva	6	6
6	Neo Balkanika EOOD	4	4	Stada Arzneimittel AG	6	6	Teva Pharmaceutical Industries Ltd	9	10	Merck Sharp & Dohme	5	10
7	Accord Healthcare	3	3	Merck Sharp & Dohme	5	11	Viatris	8	8	Polpharma Zakłady Farmaceutyczne	5	9
8	Pharmacons AD	3	3	Boehringer Ingelheim	5	5	Zydus	7	9	KrKa	5	7
9	Teva Pharmaceutical Industries Ltd	3	3	Medochemie Ltd	5	5	Merck Sharp & Dohme	6	8	Takeda Pharmaceutical Company Limited	5	6
10	Stada Arzneimittel AG	3	3	Zentiva	4	8	AstraZeneca	6	7	Teva Pharmaceutical Industries Ltd	5	5
**Top 10 MAHs**	**Romania**			**Serbia**			**Spain**					
	**Marketing authorization holder**	**No. of INNs**	**No. of Brand names**	**Marketing authorization holder**	**No. of INNs**	**No. of Brand names**	**Marketing authorization holder**	**No. of INNs**	**No. of Brand names**			

1	AstraZeneca	6	7	Hemofarm AD	6	6	Laboratorios Cinfa	9	12			
2	Merck Sharp & Dohme	5	6	AstraZeneca	6	6	Laboratorio Stada	9	9			
3	Boehringer Ingelheim	5	5	KrKa	5	6	Sandoz	8	11			
4	Gedeon Richter	5	5	Actavis	4	4	Laboratorios Normon	8	8			
5	Novartis	4	7	Boehringer Ingelheim	3	3	Aurovitas	7	8			
6	Arena Group S.A.	4	4	Galenika AD	3	3	Teva Pharmaceutical Industries Ltd	7	8			
7	KrKa	4	4	Novo Nordisk	2	3	Kern Pharma	7	7			
8	Medochemie Ltd	4	4	Berlin-Chemie AG	2	2	Astrazeneca	6	9			
9	Accord Healthcare	3	3	Sanofi	2	2	Krka	6	9			
10	Novo Nordisk	2	4	Belupo			Laboratorios Alter	6	8			

MAH= Marketing authorization holder; INN= International Non-proprietary Name

Supplementary Table S10. Top 10 Marketing authorization holders for marketed medicines

Top 10 MAHs	Bulgaria			Czech Republic			France			Poland		
	**Marketing authorization holder**	**No. of INNs**	**No. of Brand names**	**Marketing authorization holder**	**No. of INNs**	**No. of Brand names**	**Marketing authorization holder**	**No. of INNs**	**No. of Brand names**	**Marketing authorization holder**	**No. of INNs**	**No. of Brand names**
1	Tchaikapharma High Quality Medicines	5	6	AstraZeneca	5	6	Biogaran	9	11	Synoptis Pharma	3	4
2	AstraZeneca	5	5	Mylan	5	5	Arrow Generiques	9	10	Sandoz	3	3
3	Boehringer Ingelheim	5	5	Merck Sharp & Dohme	4	7	EG Labo - Laboratoires EuroGenerics	9	10	Symphar	3	3
4	Neo Balkanika EOOD	4	4	Novo Nordisk	3	4	Sandoz	8	8	Bioton	2	3
5	Medochemie Ltd	3	3	Boehringer Ingelheim	3	3	Viatris	8	8	Polpharma Zakłady Farmaceutyczne	2	2
6	Pharmacons AD	3	3	Takeda Pharmaceutical Company Limited	3	3	Zentiva	8	8	Merck Sharp & Dohme	2	2
7	Wörwag Pharma	2	2	Novatin	2	4	Teva Pharmaceutical Industries Ltd	7	8	Boehringer Ingelheim	2	2
8	Sandoz	2	2	Berlin-Chemie AG	2	3	AstraZeneca	5	5	Polfarmex S.A.	2	2
9	Actavis	2	2	Teva Pharmaceutical Industries Ltd	2	3	Cristers	5	5	Teva Pharmaceutical Industries Ltd	1	2
10	Accord Healthcare	2	2	Zentiva	2	3	Zydus	4	6	Berlin-Chemie AG	1	2

Top 10 MAHs	Romania			Serbia			Spain		
	**Marketing authorization holder**	**No. of INNs**	**No. of Brand names**	**Marketing authorization holder**	**No. of INNs**	**No. of Brand names**	**Marketing authorization holder**	**No. of INNs**	**No. of Brand names**
1	Astrazeneca	6	7	Astrazeneca	6	6	Astrazeneca	5	8

2	Arena Group S.A.	4	4	Hemofarm AD	5	5	Boehringer Ingelheim	5	5	
3	Gedeon Richter	4	4	KrKa	4	5	Cinfa	5	5	
4	Accord Healthcare	3	3	Actavis	3	3	Normon	5	5	
5	Boehringer Ingelheim	3	3	Boehringer Ingelheim	3	3	Merck Sharp & Dohme	4	11	
6	Merck Sharp & Dohme	3	3	Galenika AD	3	3	Aurovitas	4	5	
7	Novo Nordisk	2	4	Novo Nordisk	2	3	Sandoz	4	4	
8	Merck KGaA	2	3	Berlin-Chemie AD.	2	2	Stada Arzneimittel AG	4	4	
9	Sanofi	2	2	Sanofi	2	2	Takeda Pharmaceutical Company Limited	4	4	
10	Novartis and Medochemie Ltd	2	2	Belupo	2	2	Teva Pharmaceutical Industries Ltd	4	4	

MAH= Marketing authorization holder; INN= International Non-proprietary Name
